# HOXD-AS1 is a novel lncRNA encoded in *HOXD *cluster and a marker of neuroblastoma progression revealed via integrative analysis of noncoding transcriptome

**DOI:** 10.1186/1471-2164-15-S9-S7

**Published:** 2014-12-08

**Authors:** Aliaksandr A Yarmishyn, Arsen O Batagov, Jovina Z Tan, Gopinath M Sundaram, Prabha Sampath, Vladimir A Kuznetsov, Igor V Kurochkin

**Affiliations:** 1 Department of Genome and Gene Expression Data Analysis, Bioinformatics Institute, Agency for Science, Technology and Research (A*STAR), Matrix, 138671 Singapore; 2 Translational Control in Development and Disease Group, Institute of Medical Biology, Agency for Science, Technology and Research (A*STAR), Immunos, 138648 Singapore; 3 Department of Biochemistry, Yong Loo Lin School of Medicine, National University of Singapore, Singapore 117597; 4 Program in Cancer and Stem Cell Biology, Duke-NUS Graduate Medical School, 8 College Road, 169857 Singapore; 5 Division of Software & Information Systems, School of Computer Engineering, Nanyang Technological University, 639798 Singapore

**Keywords:** microarray, long noncoding RNA, neuroblastoma, biomarker, retinoic acid

## Abstract

**Background:**

Long noncoding RNAs (lncRNAs) constitute a major, but poorly characterized part of human transcriptome. Recent evidence indicates that many lncRNAs are involved in cancer and can be used as predictive and prognostic biomarkers. Significant fraction of lncRNAs is represented on widely used microarray platforms, however they have usually been ignored in cancer studies.

**Results:**

We developed a computational pipeline to annotate lncRNAs on popular Affymetrix U133 microarrays, creating a resource allowing measurement of expression of 1581 lncRNAs. This resource can be utilized to interrogate existing microarray datasets for various lncRNA studies. We found that these lncRNAs fall into three distinct classes according to their statistical distribution by length. Remarkably, these three classes of lncRNAs were co-localized with protein coding genes exhibiting distinct gene ontology groups. This annotation was applied to microarray analysis which identified a 159 lncRNA signature that discriminates between localized and metastatic stages of neuroblastoma. Analysis of an independent patient cohort revealed that this signature differentiates also relapsing from non-relapsing primary tumors. This is the first example of the signature developed via the analysis of expression of lncRNAs solely. One of these lncRNAs, termed HOXD-AS1, is encoded in *HOXD *cluster. HOXD-AS1 is evolutionary conserved among hominids and has all *bona fide *features of a gene. Studying retinoid acid (RA) response of SH-SY5Y cell line, a model of human metastatic neuroblastoma, we found that *HOXD-AS1 *is a subject to morphogenic regulation, is activated by PI3K/Akt pathway and itself is involved in control of RA-induced cell differentiation. Knock-down experiments revealed that HOXD-AS1 controls expression levels of clinically significant protein-coding genes involved in angiogenesis and inflammation, the hallmarks of metastatic cancer.

**Conclusions:**

Our findings greatly extend the number of noncoding RNAs functionally implicated in tumor development and patient treatment and highlight their role as potential prognostic biomarkers of neuroblastomas.

## Background

Whereas the coding sequences constitute only 1.5% of human genome, the noncoding constituent of the genome has recently come into light as functionally important. The most well studied part of noncoding transcriptome is represented by microRNAs (miRNAs), which affect expression of protein coding genes by modulating mRNA stability and translation. miRNAs regulate various biological processes and have well established roles in cancer [1]. However, long noncoding RNAs (lncRNAs) represent the largest fraction of transcriptional output in the mammalian genome. Unlike extensively studied protein coding transcripts or miRNAs, the majority of lncRNAs still remain "the dark matter of the genome" with their functionality being debatable. Several lncRNAs have been shown in recent years to be directly involved in oncogenesis [2]. Reprogramming of chromatin state mediated by lncRNA HOTAIR is linked to metastasis and poor prognosis in breast cancer [3]. MALAT-1 has originally been shown to be associated with metastasis in lung carcinomas [4] and regulate invasive potential of tumor cells [5].

Significant fraction of lncRNAs is represented on widely used microarray platforms, however they have traditionally been ignored in cancer studies. Thousands of microarray datasets accumulated over years in public databases provide an immense resource for analysis of lncRNA expression. In this study we set an example of reanalysis of this rich data source by annotating the noncoding probe sets of the popular Affymetrix U133 microarray and applied this annotation to identify lncRNAs associated with aggressive and nonaggressive types of neuroblastoma (NB).

NB is a common pediatric malignancy characterized by tremendous clinical heterogeneity, in which some tumors are extremely aggressive and drug resistant while others can spontaneously differentiate into benign forms and are sensitive to differentiation stimuli [6]. According to International NB Staging System (INSS), based on tumor characteristics and age, NB is classified into 3 localized stages (stages 1, 2a and 2b), advanced locoregional (stage 3) and metastatic (stages 4 and 4s) [7]. About one-half of NB patients have metastatic disease at diagnosis.

One of the most widely used prognostic genomic markers is amplification of *MYCN *locus, which occurs in 20% of NB cases. It is associated with poor survival and is considered a promising therapeutic target in NB [8,9]. All metastatic tumors with amplified *MYCN *gene are aggressive, whereas tumors with non-amplified *MYCN *gene have variable clinical behavior that could be driven by clinical (stage, histology, age at diagnosis etc) and biological factors (mutations, chromosome rearrangements, gene expression profiles).

A large number of clinical genomics and experimental model studies have described protein- coding gene expression profiles that can differentiate NBs with favorable and unfavorable outcomes [10-14]. The protein-coding gene signatures of NB aggressiveness are characterized by over-expression of genes involved in MYC and β-catenin pathways, cell cycle

and chromosome segregation, and on the other hand by low expression of genes involved in neural differentiation [15-19]. Unfortunately, reliability and consistency between the gene signatures reported in these and other publications are very poor (V.A. Kuznetsov, unpublished). It suggests high plasticity of gene expression patterns in NB tumors, a large number of correlations between potential biomarkers and high dimensionality of their space. Due to poor understanding of molecular basis of NB and high disease-caused mortality of the NB patients there is an urgent need for discovery of novel molecular drivers of NB progression and its differentiation, as well as high-confidence and reliable clinical biomarkers specifying NB tumors, prognosis of clinical outcome and improvement of NB patients' therapy.

The degree of NB differentiation correlates with stage of NB and its progression. Retinoic acid (RA) is a differentiation agent that is the first choice drug for NB therapy [20]. However, in many cases NB acquires resistance to RA treatment thus leading to relapses. Much effort has been devoted to identify biological subtypes and critical regulators of RA-induced NB differentiation. RA-induced signalling pathway in NB initiates expression of many hundreds of genes including *MYCN *and other proto-oncogenes [21]. It has also been shown to alter drug resistance mechanisms [22]. Identification of new molecular drivers, as well as unbiased, more complete and clinically relevant mechanisms of RA-induced differentiation of NB is still a great challenge.

Gain of chromosome arm 17q is one of the most common genetic abnormalities encountered in NB, which is associated with poor prognosis [23]. An example of lncRNA biomarker for NB is ncRAN gene mapped to 17q region [24]. Its over-expression was shown to be associated with aggressiveness of primary NB [24] and confer a set of oncogenic properties, such as acceleration of cell growth and increasing invasiveness [24,25]. One of the classes of highly conserved lncRNAs, known as transcribed ultraconserved regions (T-UCR) has been shown to be implicated in cancer [26]. Indeed, differential expression of T-UCRs was shown to correlate with such oncogenomic parameters as *MYCN *amplification status and be a consequence of DNA copy number changes associated with NB [27]. Another study revealed association of different sets of T-UCRs with poor and long-term survival in the metastatic stage of NB patients [28]. In spite of this evidence for involvement of lncRNAs in NB, systematic and unbiased genome-wide analysis has not been performed.

In this study, we performed systematic genome-wide analysis to identify biomarkers for aggressive stages of NB. For these purposes we took advantage of the extensive coverage of a considerable part of noncoding transcriptome by Affymetrix U133 Plus 2.0 microarray probe sets. These probe sets have traditionally been neglected in most studies aimed to identify tumor biomarkers. Here, we developed a computational pipeline to annotate noncoding probe sets on Affymetrix U133 chips, representing ESTs, non-annotated RNAs, and unknown ncRNAs and identified lncRNA transcripts that differentiate favorable from aggressive stages of NB, as well as relapsing and non-relapsing primary tumors. We found that one of the identified in this study lncRNA transcript, termed HOXD-AS1, is induced by RA, regulated by PI3K/Akt pathway and controls genes implicated in RA-mediated cell differentiation and also angiogenesis and inflammation, the hallmarks of metastasized cancer [29].

## Results

### Affymetrix U133 series microarrays contain 1,586 probe sets measuring expression of potential lncRNAs, including 549 high-confidence lncRNA transcripts

**Figure 1:**
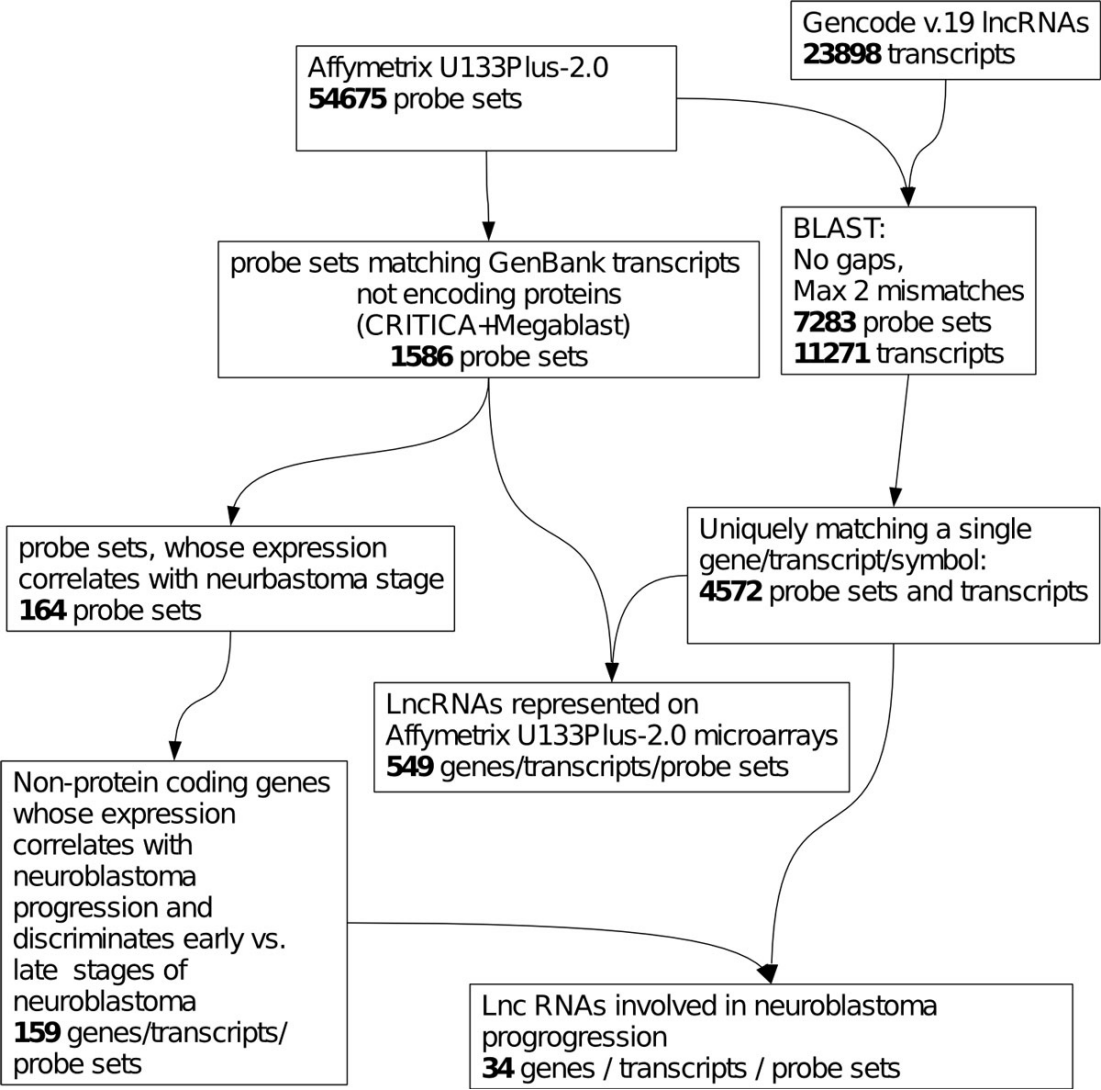
**Pipeline for the automated annotation of Affymetrix U133 lncRNAs**. Blocks represent data sets emerging from the analysis and rules used for their generation. Numbers represent the number of entries in each dataset.

The probe sets of Affymetrix U133 chips were designed to detect not only transcripts of protein-coding genes, but also many transcripts currently annotated as ESTs and non-annotated RNAs(30), some of them can measure expression of unknown lncRNAs. Our goal was to find such probe sets. The 54,675 probe sets of Affymetrix microarrays U133A, U133B and U133Plus-2.0 were subjected to the filtration by quality and uniqueness of transcript matching according to the criteria of APMA database described by us earlier [30] (Figure 1). Following the database annotation, 7,587 probe sets matching unique genomic locations of RNAs not annotated as protein mRNAs were selected. Probe sets overlapping with exons of any known proteins (RefSeq) on the same DNA strand were excluded from the analysis. Thus, 1,586 probe sets were selected (Additional file 1), each of them matched a single transcript. To ensure that the selected transcripts do not encode proteins, we analyzed them with CRITICA software that identifies protein-coding sequences by potential presence of codons in the transcript sequences [31,32]. All 1,586 transcripts passed this filter and, therefore, they were considered as probe sets potentially measuring expression of lncRNA genes. To find which of these genes can be considered as true lncRNA candidates, their length distribution was analyzed. To ensure the reliability of the results, the sequences of individual Affymetrix probe sets were independently scanned against the 23,898 sequences of lncRNA transcripts annotated by Gencode consortium (v.19) using NCBI BLAST tool. Only 4,572 transcripts were found to have uniquely matching probe sets, 549 of which were present among our 1,586 ncRNA candidates. Since there was a limited overlap between the candidates and the Gencode-annotated lncRNAs, we considered the 549 lncRNAs as a high-confidence subset embedded in our data.

### Length distribution of the ncRNA candidate transcripts reveals three lncRNA classes

**Figure 2:**
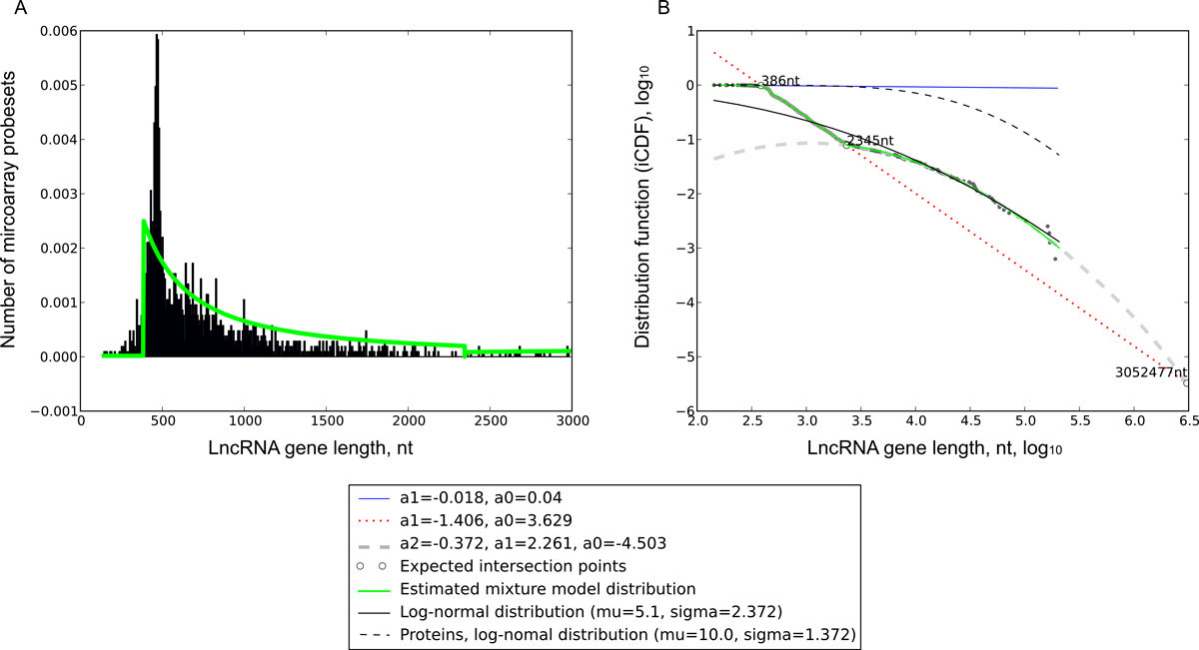
**Length distribution reveals three classes of ncRNAs**. (A) Histogram of length distribution of all ncRNAs represented on AffymetrixU133 arrays; solid gray line represents the proposed three-component mixture model. (B) Inverse cumulative distribution (iCDF) of the ncRNA length plotted in double log coordinates reveals the three components of the mixture model represented as solid, dotted and dashed gray curves respectively; each component is parametrized with a polynomial equation (coefficients a0, a1 and a2); log-normal distribution fitting only the third component of the mixture model is represented with black solid line.

Traditionally, ncRNAs longer than 200 nt are considered lncRNAs. According to this criterion, 1,581 selected ncRNAs were classified as lncRNAs. The distribution of the lncRNAs by length can be described as a mixture of three groups (Figure 2). Two distributions with very long and moderate long sequence lengths were fitted well with power-law like functions [33,34]. The length distribution of relatively shorter 3-rd group of lncRNAs resembles a log-normal distribution whose shape is more similar to the frequency distribution of protein-coding genes (Figure 2B, Additional file 2). The expected lncRNA lengths marking the three ranges correspond to each of the three distributions. They were found from the extrapolation of the components' estimates to their intersection points giving lengths of 386 nt and 2,345 nt. Thus, based on these cutoff estimates, we propose the following groups of lncRNA genes: 1) lncRNAs-1, length from 200 nt to 386 nt, 2) lncRNAs-2, length from 386 to 2,345 nt and 3) lncRNAs-3, length >2,345 nt. These classes comprise approximately 4%, 87% and 9% of 1,581 selected lncRNAs.

### Genes encoding lncRNA-2 and lncRNA-3 classes are localized next to transcription factor genes implicated in development

Since the vast majority of the studied lncRNAs have never been functionally classified, we analyzed the functions of their neighboring genes (within +/-10 kb distance). Due to their proximity, they may be co-regulated with the lncRNA genes and thus may suggest biological processes associated with these genomic regions. For 89% (1400/1581) of lncRNAs such neighbors were found. For the classes lncRNA-1, lncRNA-2 and lncRNA-3 this fraction was 89% (62/70), 89% (1218/1372) and 93% (129/138), respectively (Additional files 1 and 3). Gene ontology (GO) analysis of all lncRNAs showed significant enrichment for 13 ontologies, including 'Wnt pathway', 'nucleic acids metabolic processes', 'mitochondria' and 'transcription factors' (Additional file 4). Out of four GO terms associated with lncRNA-1, three ('lysosomal transport', 'hydrolase activity of N-glycosyl compounds', 'phosphodiesterase') were common with all lncRNAs. In contrast, class lncRNA-2 was associated with 10 GO terms common with all lncRNAs. 'Homeobox transcription factors' and 'gut mesoderm development' categories were absent in lncRNA-2 class GO terms and were unique for lncRNA-3 class. Class lncRNA-3 was significantly associated with 15 GO terms, i.e. more than for all lncRNAs. GO terms 'reproduction', 'embryonic development' and 'KRAB box transcription factors' were associated only with lncRNA-3 class. It is remarkable that among the neighbors of lncRNA-3 class genes 'gut mesoderm development' category was represented by 11 genes from 3 *HOX *clusters: *HOXA, HOXB*, and *HOXD*. These transcription factors constitute 88% of all 18 lncRNAs annotated with this GO term. Two other homeobox genes, not belonging to *HOX *were *EMX2 *and *MNX1*. Fifteen other transcription factor genes belong to the zinc finger class and the remaining 8 genes to other classes.

### 159 lncRNAs are associated with NB progression

**Figure 3:**
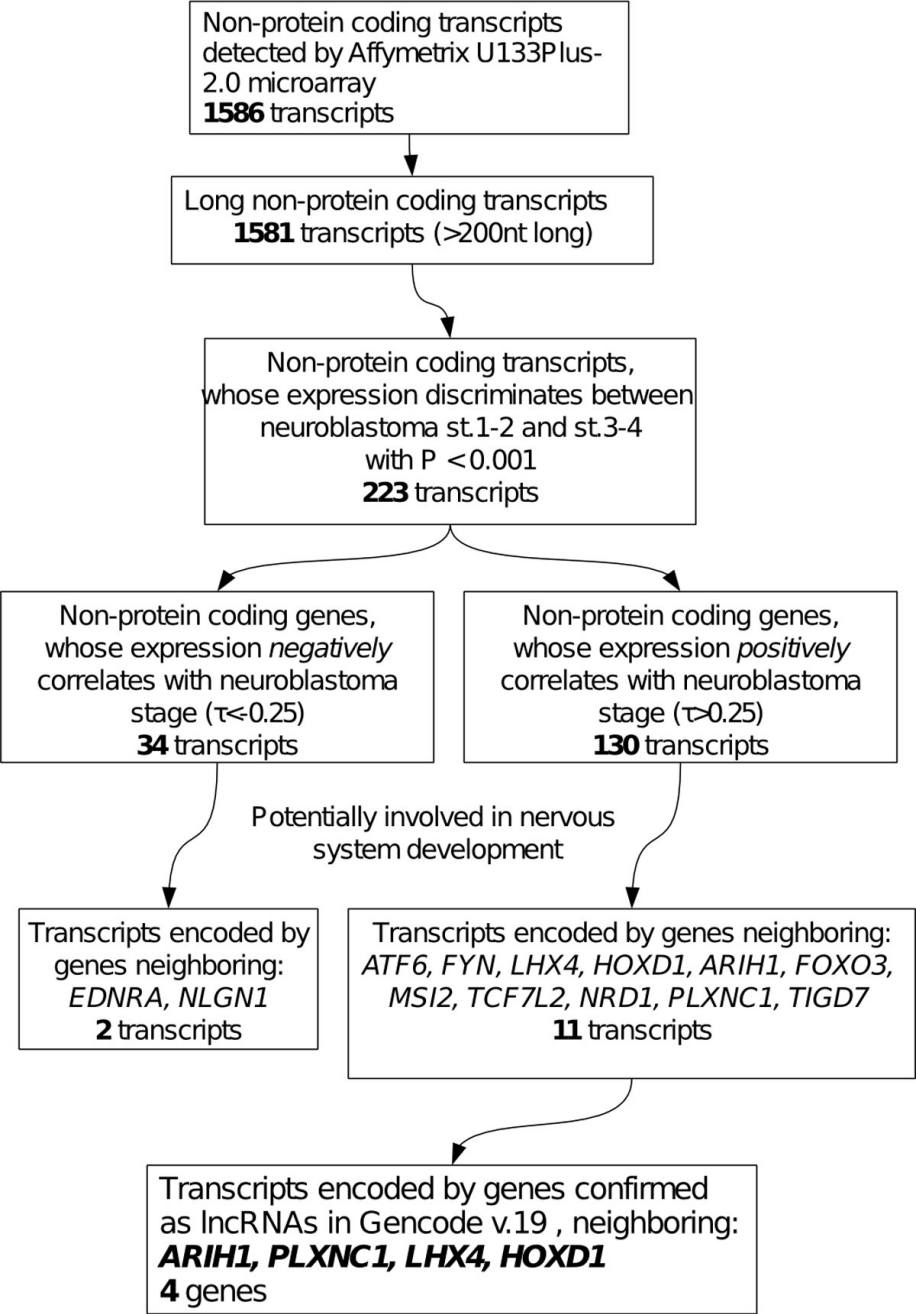
**Pipeline for identification of lncRNA biomarkers that discriminate between stages 1-2 and 3-4**. Blocks represent data sets emerging from the analysis and rules used for their generation. Numbers represent the number of entries in each dataset.

Data from NB patient tumor samples (GSE12460) were used to make a primary screening of lncRNAs associated with NB. Fourteen tumors in this dataset were MYCN-amplified (MNA), while the rest 50 tumors had no MYCN amplification (non-MNA). 1,581 lncRNAs represented on Affymetrix microarray U133Plus-2.0 were analyzed for their association with the stages of NB progression in the non-MNA group of patients (Figure 3). After comparing expression levels of these genes in early stage (1 and 2) vs. late stage (3 and 4) tumors 223 of them were identified as genes whose expression could discriminate (P < 0.001) between the stages. Among them 164 lncRNAs, whose expression levels significantly correlate (Kendall's |τ| > 0.25) with the stage were found (Additional file 5A). Among them 79% (130/164) correlated positively. PAM analysis revealed that 159 of the 164 lncRNAs form a gene signature able to discriminate between the low-aggressive (stages 1, 2) and high-aggressive (stages 3, 4) tumors with 79-88% accuracy (Additional files 5B and 5C). This signature also discriminates between relapsing and not relapsing primary tumors (GSE3446) with 74-80% accuracy (Additional files 5D and 5E). The lncRNAs of this tumor aggressiveness/relapse gene signature are grouped in two clusters by their correlation coefficients (Additional files 5F and 6). Analysis of GO of the genes neighboring the 126 lncRNAs of the main cluster demonstrated a significant enrichment in genes associated with nucleic acid binding and transcription (Additional file 5G).

Importantly, this list of lncRNA candidates included 13 genes (including four high-confidence lncRNAs) whose neighboring protein-coding genes were annotated as involved in embryonic development (Figure 3). Studying them could give a clue for the mechanisms of lncRNA involvement in neuroblastoma progression. Therefore, we tested the expression of each of the 13 lncRNAs in response to the treatment of neuroblastoma cells to RA. RA is a morphogen, which can induce neuronal differentiation in embryo and cell culture and is widely used for therapy of neuroblastoma.

### HOXD-AS1 is up-regulated by retinoic acid in SH-SY5Y neuroblastoma cell line

**Figure 4:**
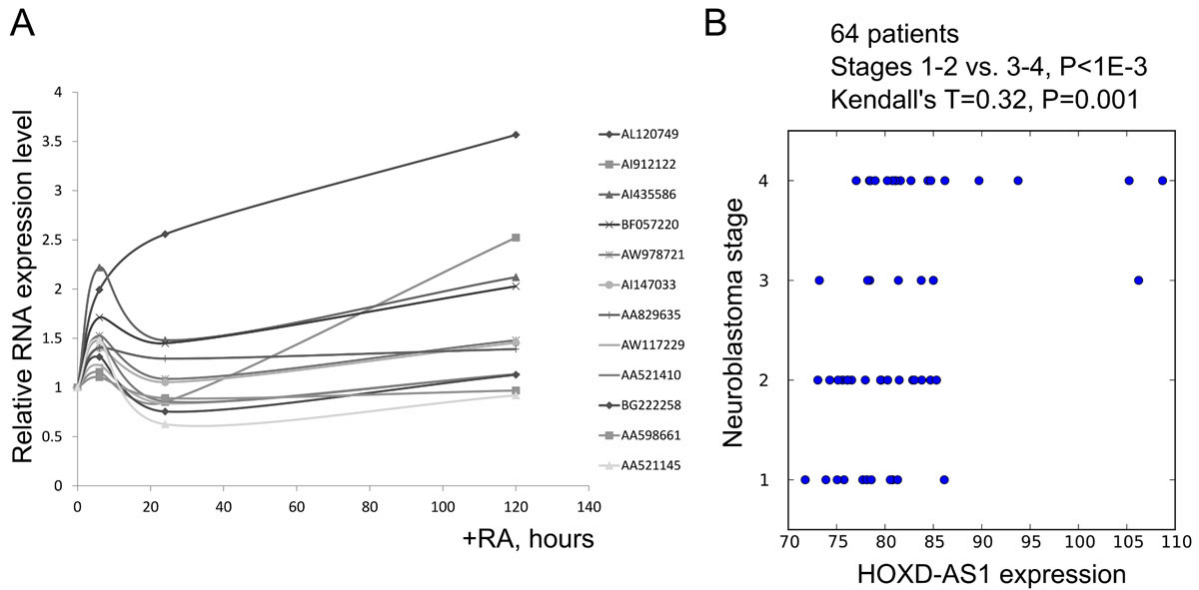
**Up-regulation of HOXD-AS1 in SH-SY5Y neuroblastoma cell line by RA and stage correlation in patients**. (A) SHSY5Y cells plated onto laminin coated dishes were induced to differentiate with 10 µM retinoic acid (RA). Total RNA was extracted at the time-points of 0 hours (uninduced), 6, 24 and 120 hours. The amounts of the selected 13 probes were quantified by qRT -PCR using relative DDCt quantification method and expressed as a fold change relative to uninduced time-point (0 hours). (B) HOXD-AS1 expression correlates with neuroblastoma progression and differentiates between early (st. 1-2) and late (st. 3-4) stage tumors. Each patient of the whole studied population of 64 patients diagnosed with NB (55) is represented by a dot.

In our experiments, we chose non-MNA neuroblastoma cell line SH-SY5Y, which is a widely used model for RA-induced neuronal differentiation. In our recent work, we performed analysis of differential expression of the 13 lncRNAs upon differentiation of SH-SY5Y cells using custom microarray chip [35]. Among all 13 tested lncRNAs only one lncRNA was upregulated during differentiation [35] was represented among the 164 lncRNA genes, correlating with NB stage, measured in patient tumors by a probe set 239182_at. Using qRT-PCR we measured the expression of these 13 lncRNAs at four time points (0 hr, 6 hr, 1 day and 5 days) post RA induction. The expression of only one transcript AL120749, encoded by gene HOXD-AS1, was progressively up-regulated by RA up to the highest level at the 5th day after RA-induction (Figure 4A). It is located between *HOXD1 *and *HOXD3 *genes in anti-sense direction to both of them. Noncoding status of HOXD-AS1 was predicted by both Megablast and Critica algorithms (31). Interestingly, expression level of probe set 239182_at correlates with the progression stage of the NB patients (τ = 0.32, P = 0.001) (Figure 4B), including non-MNA (τ = 0.28, P = 0.01) patients. It also discriminates the tumors at stages 1-2 from the tumors at stages 3-4 (P < 0.001), including non-MNA (P = 0.009) and MNA (P = 0.002) patients (Additional file 7).

### Characterization of HOXD-AS1 locus and its transcripts

**Figure 5:**
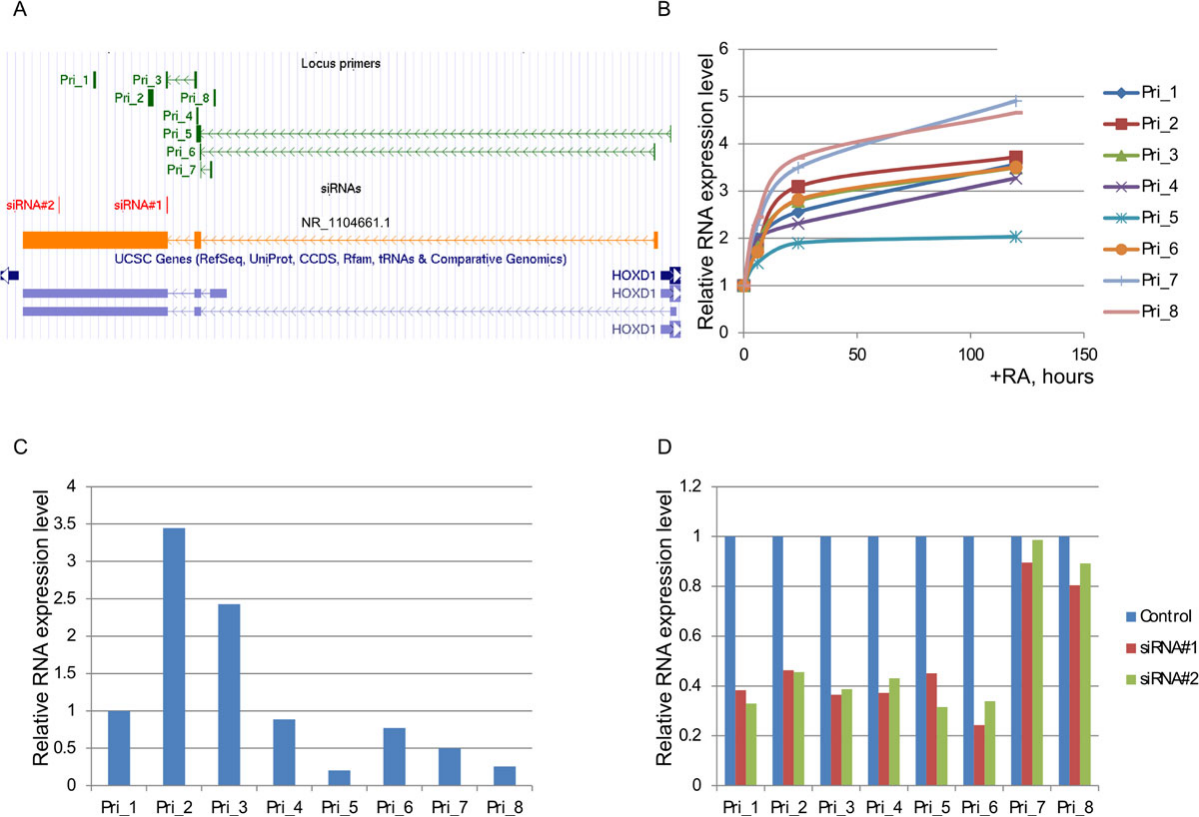
**Characterization of HOXD-AS1 transcript**. (A) Positions of different primer pairs targeting different annotated transcripts in the intergenic locus between *HOXD1 *and *HOXD3 *genes (Locus primers track, green) mapped in UCSC browser relative to HOXD-AS1 RefSeq NR_1104661.1 (orange) and UCSC Genes track LOC401022 transcripts (blue). (B) SH-SY5Y cells were induced to differentiate with RA and total RNA was extracted at the time-points of 0 hours (uninduced), 6, 24 and 120 hours. The relative levels of the different transcripts amplified by the indicated primer pairs were measured by qRT-PCR and expressed as fold change relative to the uninduced (0 h) time-point. (C) The relative expression levels of the indicated target transcripts in uninduced SH-SY5Y cells were measured by qRT-PCR. (D) Effect of siRNA#1 and siRNA#2 (see Materials and Methods) on the relative levels of various parts of HOXD-AS1 transcript as determined by qRT-PCR.

Within the locus between *HOXD1 *and *HOXD3 *genes several isoforms of HOXD-AS1 have been annotated with two transcription start regions defined by active promoter features, such as CpG islands, H3K27 acetylation and transcription factor ChIP tracks (Additional file 8). The probe set 239182_at, which discriminates the patients, at its 5'-end fits into the boundary of RefSeq transcript NR_1104661.1, which starts at the promoter region 1. In order to define the boundaries of HOXD-AS1 transcript in SH-SY5Y cells, a set of primer pairs targeting different possible exons within this region was designed (Figure 5A, Additional file 8). Treatment of SH-SY5Y cells with RA resulted in induction of all regions under query with a 3-5-fold magnitude for most of them, apart from the primer pair 5 (Pri_5) querying the first two exons of UCSC LOC401022 isoform starting at the promoter region 1, which was induced only 2-fold (Figure 5B). The comparison of the relative levels of different amplicons revealed that the least abundant was UCSC LOC401022 isoform starting at the promoter region 1 (Pri_5), Pri1 - Pri4 querying regions common for all possible transcripts were of the highest abundance and Pri_6 querying exons 1-2 of NR_1104661.1 was of comparably high level (Figure 5C). The knock-down by two siRNAs used in this study as described below (their target positions shown in siRNAs track of Additional file 8) resulted in depletion of regions fitting to the RefSeq NR_1104661.1 (Pri_6) and the UCSC Genes LOC401022 isoform starting at promoter region 1 (Pri_5), but not the UCSC Genes LOC401022 isoform starting at the promoter region 2 (Pri_7 and Pri_8, Figure 5D). In summary, RefSeq NR_1104661.1 transcript was the most abundant in SH-SY5Y cells and the most responsive to experimental conditions of RA-induced differentiation and siRNA mediated knock-down. Although the probe set 239182_at features the shortened form of the exon 3 as compared to HOXD-AS1 annotated transcripts, the pairs of primers spanning its entire length as in the UCSC geneset **("Exon 3 primers" track**, Additional file 8) produce comparable relative qRT-PCR signals (Additional file 9**panel A**) and are depleted by both siRNAs (Additional file 9**, panel B**), implying that the full length of this exon is present in the transcript under query. Indeed, the predicted full length size of NR_1104661.1 transcripts of 3,821 nucleotides is confirmed by Northern blot using the probe targeting exon 3 (Additional file 9**panel C**).

### Evolutionary analysis of *HOXD-AS1 *gene

**Figure 6:**
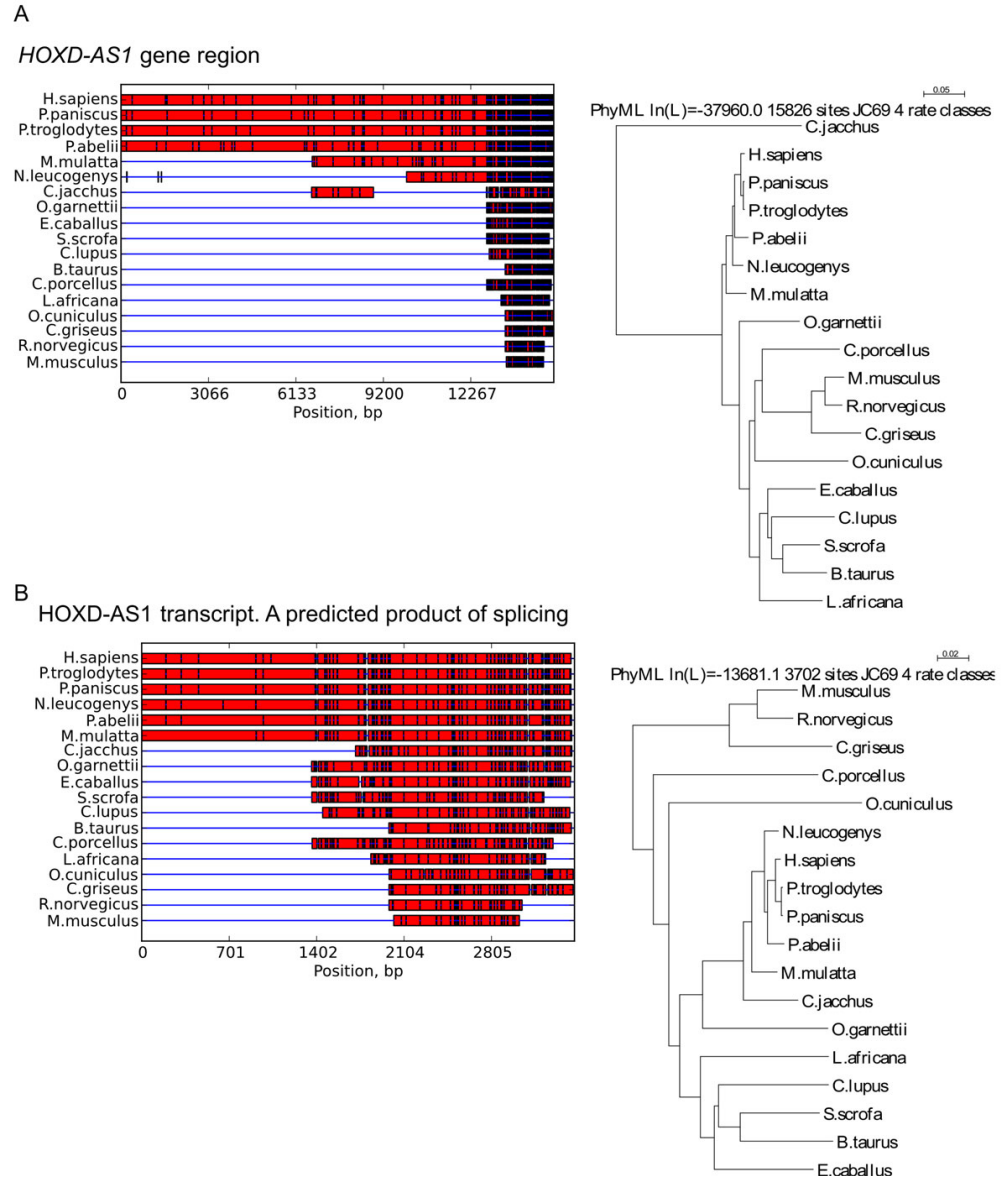
**Evolutionary analysis of HOXD-AS1 transcript**. (A) The 3'-region of *HOXD-AS1 *gene is conservative among mammals, while the middle part shares conservation in primates and the 5'- region in hominids only. (B) The putative spliced variant of the major HOXD-AS1 transcript is conservative among primates, while its 3'-part is common for mammals.

A comparison of *HOXD-AS1 *gene region against NCBI database of reference genomic sequences with BLAST showed that more than 96% of this gene sequence is evolutionary conserved among hominids (*H. sapiens, P. paniscus, P. troglodytes *and *P. abelii*). At the same time, only 12% to 52% of *HOXD-AS1 *sequence is shared with other primates *(M. mulatta, N. leucogenys, C. jacchus *and *O. garnettii*), comparable to 11% of dog (*C. lupus*) and 8-12% of ungulates (*E. caballus, S. scrofa *and *B. taurus*) (Figure 6). Thus, a gap between *HOXD-AS1 *genomic sequence in apes and other monkeys is apparent.

### Tissue expression pattern of HOXD-AS1 correlates with *HOXD1 *and *HOXD3 *genes expression

**Figure 7:**
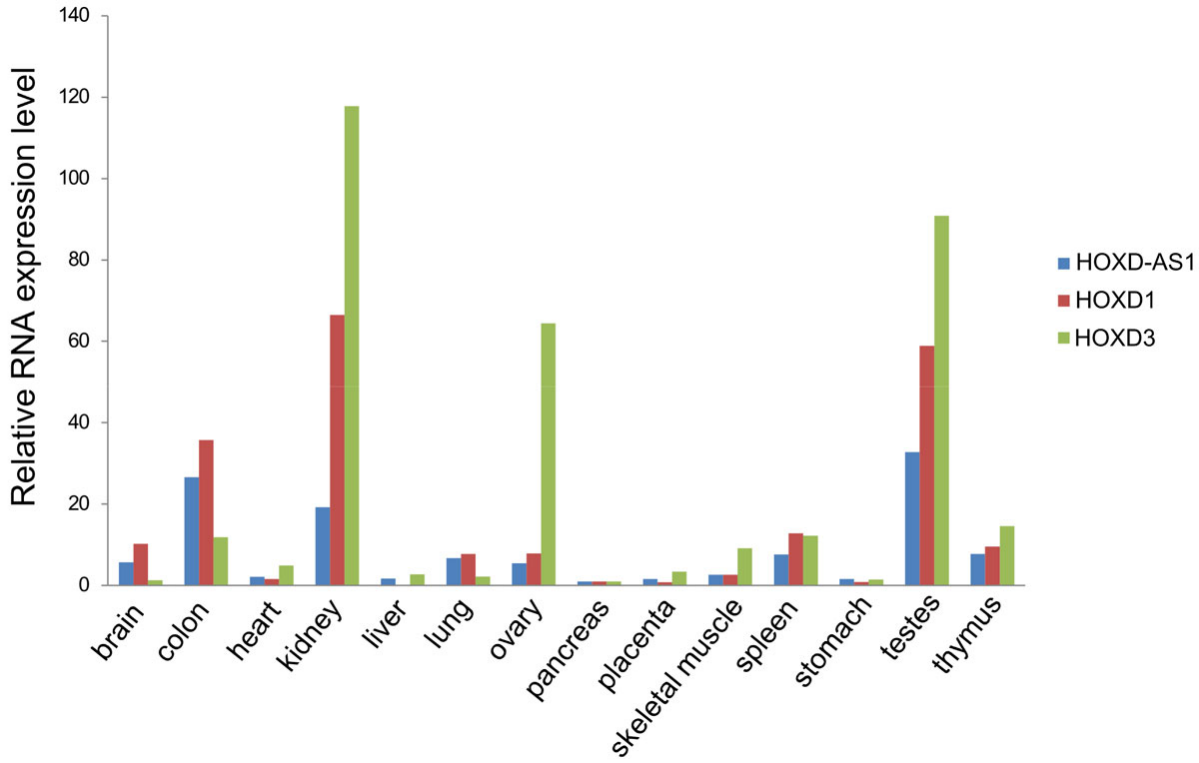
**Tissue expression pattern of HOXD-AS1 correlates with HOXD1 and HOXD3 transcripts**. The amounts of HOXD-AS1 transcript and adjacent HOXD1 and HOXD3 mRNAs in human organ panel (Origene) were analyzed by qRT-PCR. The data are expressed as a proportion to the amount of the respective transcripts in pancreas.

We characterized the pattern of expression of HOXD-AS1 in human body by qRT-PCR analysis of human organ cDNA panel. We found that HOXD-AS1 is expressed in multiple tissues with the highest levels of expression in kidney, colon and testes and the lowest in liver, heart, pancreas and stomach (Figure 7). In general, HOXD-AS1 demonstrated highly correlated expression with its neighboring protein coding genes HOXD1 and HOXD3, thus implying that they are subject to the common regulatory mechanism. Notably, in the non-MNA patient tumors HOXD-AS1 expression significantly correlated with HOXD3 (τ = 0.25, P = 0.007) (Additional file 10), but not HOXD1.

### Differentially expressed genes identified after knock-down of HOXD-AS1 in SH-SY5Y cells

**Figure 8:**
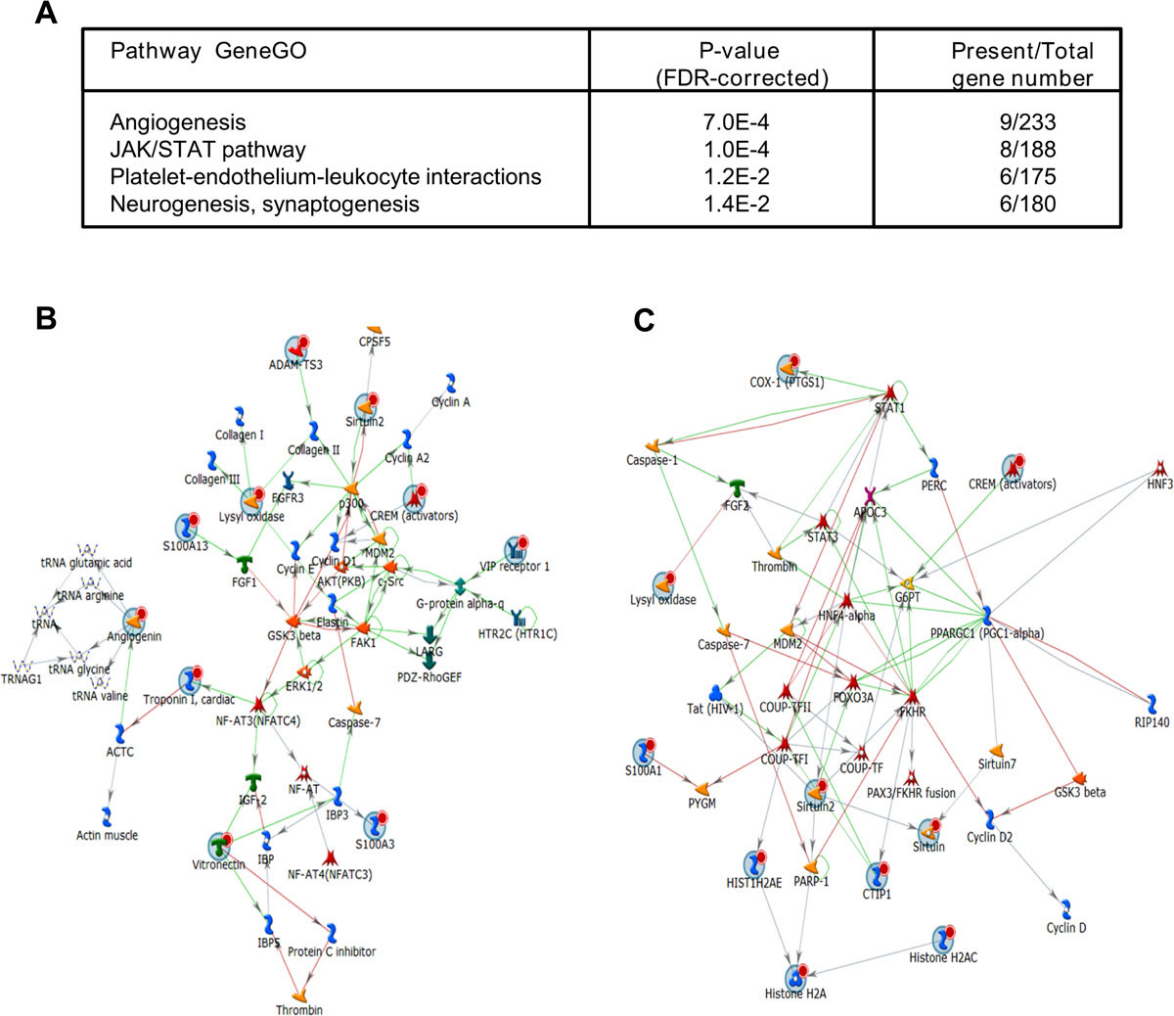
**Significantly overrepresented pathways identified from genes regulated by HOXD-AS1**. The genes, whose expression is regulated by HOXD-AS1 are represented by four MetaCore process networks with significant enrichment. (A) The list of pathways with significant enrichment. (B) A part of angiogenesis process network including HOXD-AS1 target genes (marked with red dots). (C) A part of JAK/STAT process network including HOXD-AS1 target genes (marked with red dots).

The differential expression of HOXD-AS1 in NB patients and in differentiating NB cell line is an indicator of functionality of this lncRNA. In order to gain more insight into functionality of HOXD-AS1 we sought to investigate its effect on global gene expression profile in the context of differentiation of SH-SY5Y cells. For this purpose we designed two siRNAs targeting different regions of HOXD-AS1. Both of these siRNAs knocked down HOXD-AS1 transcript in the RA-induced SH-SY5Y cells with 70 - 80% efficiency as compared to non-targeting siRNA control (Additional file 11A). The total RNA was hybridized to IlluminaBeadArray (deposited in NCBI GSE40680) and differentially expressed genes common for two siRNAs were identified. In total, we identified 96 differentially regulated genes, among them 43 were up-regulated and 53 were down-regulated (Additional file 12). The pattern of differential expression of a set of genes, most of which were chosen for their involvement in the processes of inflammation and angiogenesis as discussed further, was confirmed by qRT-PCR (Additional file 11B). In order to annotate the functions of differentially expressed genes at the network level we mapped them on existing networks using MetaCore pathway analysis software. We observed significant functional enrichment in pathways regulating angiogenesis and inflammatory JAK/STAT pathway (Figure 8).

### Expression of genes controlled by HOXD-AS1 significantly correlates with its expression in clinical neuroblastoma samples

In non-MNA tumors we analyzed pair-wise correlation between expression of HOXD-AS1 and of its targets revealed in the knock-down experiments. Among the genes regulated by HOXD-AS1 in the cell line, 53 were represented on the Affymetrix chip by 98 probe sets. The expression of 21 of them significantly (P < 0.05) correlated with HOXD-AS1expression in the NB samples (Additional file 12). Sixteen genes correlated positively and 5 negatively. Expression of 20 HOXD-AS1 targets was significantly different in primary tumors of 102 patients with relapse (P < 0.05), seven of which at the same time correlated with NB stage (Additional file 12). Importantly, *MAGEA9B *gene expression was also a significant (P = 0.014) marker of 5-year survival of neuroblastoma patients (GSE16237). Additionally, *MAGEA9B*, along with two other targets of HOXD-AS1 - TNF and CNR1, were recently found to be a part of neuroblastoma signature identified in several datasets [36]. Sixteen of HOXD-AS1 target genes were predictive markers of NB relapse (Additional file 13). Remarkably, 12 out of these 16 genes were previously reported to respond to RA treatment (Additional file 14A). Among these genes *SNN, TMEM86A, VIPR1, CREM *were down-regulated and *TSPAN2, CNR1, CREBL1, PTGS1 *were up-regulated by RA. Gene signature consisting of four HOXD-AS1 targets, *ADAMTS3, AMDMD2, ANG, ASNA1 *could predict NB relapse with 76-80% accuracy (Additional files 14B and 14C). Thus, HOXD-AS1, in response to RA treatment, controls expression of genes involved in NB progression. Some of them can serve as predictive markers of NB relapse and are associated with patient survival.

### HOXD-AS1 is evenly distributed between nucleus and cytoplasm

So far most of the characterized lncRNAs that effect gene expression are known to be localized to the nucleus. Therefore, we analyzed the subcellular localization of HOXD-AS1. qRT-PCR analysis of subcellular fractions of SH-SY5Y cells revealed that HOXD-AS1 is equally distributed between the nucleus and the cytoplasm (Additional file 15A). This pattern was largely confirmed by RNA fluorescence *in situ *hybridization. HOXD-AS1-specific probe sets detected individual transcripts both in the nucleus and the cytoplasm (Additional file 15B).

### HOXD-AS1 expression is regulated by PI3K/Akt signaling pathway

**Figure 9:**
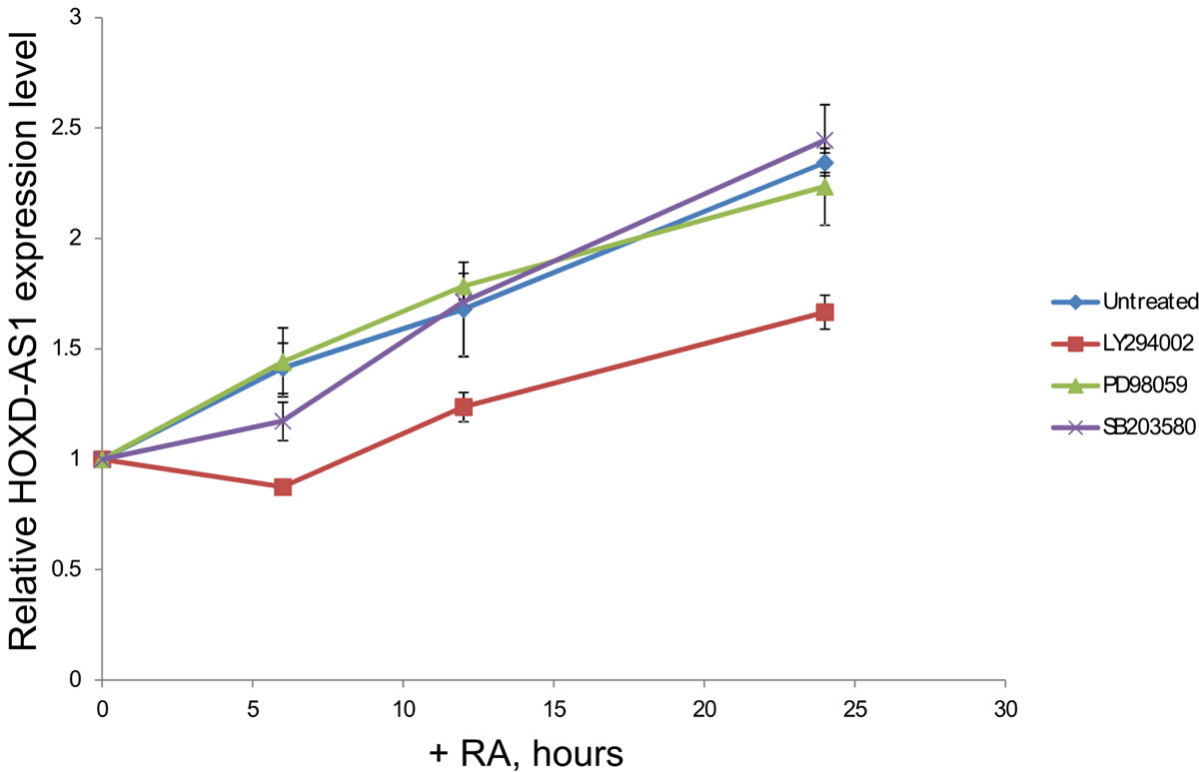
**HOXD-AS1 expression is regulated by PI3K signaling pathway**. SH-SY5Y cells were induced to differentiate with 10 µM retinoic acid (RA) in the absence or presence of 10 µM LY294002 (inhibitor of PI3K), PD98059 (inhibitor of MEK1) and SB203580 (inhibitor of p38-MAPK). RNA was extracted in the time-course of 6, 12 and 24 hours and the fold change of induction of HOXD-AS1 was estimated by qRT-PCR relative quantification analysis. Each treatment for each time-point was performed in triplicate with standard deviation error bars shown as indicated.

Induction of differentiation of NB cells by RA is a complex process, requiring involvement of at least two signaling pathways: PI3K/Akt and MAPK/ERK [37]. To characterize regulation of expression of HOXD-AS1 by these signaling pathways we used chemical inhibitors of PI3K (LY294002), MEK1 (PD98059) and p38-MAPK (SB203580). SH-SY5Y cells were induced to differentiate in the presence of these inhibitors in the time course of 6, 12 and 24 hours and change of HOXD-AS1 expression was measured by qRT-PCR. The induction of HOXD-AS1 was significantly reduced in the presence of LY294002, however it was not affected by either PD98059 or SB203580, thus indicating that HOXD-AS1 is regulated by PI3K/Akt pathway, but not by MAPK/ERK (Figure 9).

## Discussion

Evidence for involvement of different lncRNAs in oncogenesis and for their potential use as biomarkers of cancer is growing [2,38]. Affymetrix U133 series are among the most widely used microarray platforms with thousands of datasets deposited in the public databases. Given the fact that many lncRNAs were fortuitously represented on this platform these datasets provide a great potential for their reanalysis, with lncRNAs taken into account. Here we annotated the noncoding fraction of Affymetrix probe sets and created a resource for this kind of studies.

Discrimination of coding and non-coding transcripts is not straightforward, and different laboratories are using different methods. Therefore, in our work we used two different methods that discard the possibility of any transcript encoding a protein: Megablast, to eliminate the possible known protein homologs; followed by CRITICA to eliminate the possibility that any of our transcripts may encode a protein, yet to be discovered [31,32]. All 1,586 transcripts studied in our work satisfied both criteria. In addition to that, we applied the criterion of transcript length distribution among proteins to judge about our dataset as a whole. To maximize the reliability of prediction, we performed an additional analysis and mapped the probe sequences to the lncRNA transcript sequences predicted by Gencode consortium. It allowed us to identify 549 reliable probesets corresponding to lncRNAs out of 1,586 initially predicted. We provided classification of these 1,586 lncRNAs by the length of their transcripts and discriminating three classes among the lncRNA genes longer than 200 nt. Our classification system is vindicated by the fact that the proposed lncRNA classes have distinct gene ontologies.

However, lncRNA prediction strategy utilized in this study does not take into account relative position of candidate lncRNA gene in relation to protein coding gene. It has been a prevalent belief for some time that intronic parts of mRNAs cannot be considered as lncRNAs because they merely represent pre-mRNAs on their way to splicing or spliced-out introns on their way to degradation. The lower expression of the intronic regions than those in exons seemingly was consistent with this notion. However, several recent studies have focused on this important issue and concluded that intronic lncRNAs have a number of features that would be expected from functional, stand-alone RNA species. For example, St Laurent et al. have shown that there are thousands of intronic RNAs in the mouse genome changing throughout the inflammation time course that are likely to be functional [39]. The levels of intronic RNAs were independent of those of exons or other introns and some of the introns encoded relatively abundant RNA species. Another study by Tahira *et al*. described intronic lncRNAs differentially expressed in primary and metastatic pancreatic cancer [40]. The authors suggested that intronic lncRNAs are generated from independent transcriptional units. In support of this notion they have shown enrichment of H3K4me3 marks close to the known boundaries for the set of intronic transcripts, the presence of conserved DNA elements and stable predicted secondary structures in intronic lncRNAs, evidence for the evolutionary selection, and significant enrichment of CAGE tags proximal to known start sites of intronic lncRNAs [40].

Applying the new probe set and ncRNAs annotation system for biomarker discovery in public datasets, we identified a gene expression signature consisting of 159 lncRNAs, which can discriminate NB tumors by their aggressiveness and clinical stages. To our knowledge, this is the first example of a gene signature based on expression of lncRNAs only. Functional studies of lncRNAs are not common and the biological effects of this class of molecules have not been studied well. The primary structures of lncRNAs are much less conservative in comparison with protein structures. Therefore, functional characterization of human lncRNAs is particularly difficult. Characterization of lncRNAs via gene signatures specific to certain diseases reported in the present study can be used as a tool to solve this methodological conundrum. It is important that our syndrome-centered scan for potential candidates was successfully validated and complemented with mechanistic studies on a model cell line. Regarding the clinical potential of gene expression signatures based only on lncRNAs, it is important to note that such signatures can be particularly useful as diagnostic and prognostic tools in combinations with existing protein-based signatures, since lncRNAs and proteins belong to different regulatory realms and thus naturally increase each other's power as patient classifiers.

Among the 159 lncRNAs we selected HOXD-AS1 for functional characterization as the most potent candidate for playing a role in oncogenesis. The gene encoding HOXD-AS1 is located in *HOXD *cluster. *HOX *genes are the key developmental regulators and their aberrant expression is often associated with malignancy [41] . We obtained evidence of a two-step evolution of the unspliced *HOXD-AS1 *sequence. The first step, characterized with initial two-fold increase of *HOXD-AS1 *length, presumably occurred around the time of primates branching from other placental animals between 115 [42] and 80 million years ago [43]. The second step, when the maximal-length *HOXD-AS1 *gene in hominids (*H. sapiens, P. paniscus, P. troglodytes and P. abelii*) was established, might have occurred 15-19 million years ago, when hominid diverged from hylobatids (*N. leucogenys*) [44].

Remarkably, *HOXD3 *gene, adjacent to *HOXD-AS1 *locus, has established role in promoting metastatic potential of lung cancer cells [45]. *HOXD1*, which is also adjacent to *HOXD-AS1*, has been linked with locus rs2072590 associated with an increased risk of ovarian cancer, more significantly for the serous subtype [46]. This locus lies in a non-coding region, ~5 kb distal to *HOXD3 *and ~10 kb proximal to *HOXD1 *[46]. Using higher resolution map, we found that SNP rs2072590 is localized in *HOXD-AS1 *and includes the last intron and last exon regions boundary.

Interestingly, differential expression of HOXD-AS1 lncRNA was detected by microarray analysis in a number of cancer studies. It was among the 17 genes up-regulated in EGFR-mutated adenocarcinomas, which are characterized by poor post-operative prognosis [47]. In breast cancer, over-expression of breast cancer metastasis suppressor gene (BRMS) leads to down-regulation of HOXD-AS1 [48], thus further pointing to its potential pro-oncogenic role. In contrast, HOXD-AS1 was shown to be one of the most drastically down-regulated genes in hepatocellular carcinoma [49]. We also found that HOXD-AS1 is strongly suppressed in colorectal adenoma discriminating these precancerous lesions from the surround normal mycosa tissue [50]. Here we show that HOXD-AS1 is highly induced by RA treatment of NB SH-SY5Y cell line. RA causes growth arrest and differentiation of NB cells, therefore it is widely used for therapeutic purpose. The elevation of HOXD-AS1 levels in metastatic tumors is in a seeming contradiction with its up- regulation during tumor cell differentiation. One possible explanation is that HOXD-AS1 elevation in metastatic tumors is a compensatory reaction to a loss of its differentiation-promoting function that actually leads to aberrant tumor-promoting effect. This could happen because of the overall loss of homeostasis when inactivation of one pathway leads to a compensatory activation of another pathway.

In this study, we investigate regulation of *HOXD-AS1 *expression within a framework of PI3K and MAPK signaling pathways, both of which are necessary for RA mediated differentiation and survival of SH-SY5Y cells [37]. Here we demonstrate that chemical inhibitor of PI3K, but not inhibitors of components of MAPK pathway MEK1 and p38 significantly reduce RA-mediated induction of HOXD-AS1 (Figure 9). Both PI3K and MAPK pathways are induced in SH-SY5Y cells by ligation of a family of growth factors, known as neurotrophins to their respective receptors belonging to a class of receptor tyrosine kinases. Different types of neurotrophin receptors are known to control differentiation and survival of neuroblastoma cells include TrkA, TrkB, TrkC [51]. Whereas TrkA and TrkC are not regulated, or regulated marginally by RA in SH-SY5Y, TrkB is efficiently induced by it (Additional file 16**panel A**). Interestingly, activation of PI3K pathway by ligation of TrkB with its specific ligand BDNF leads to both neuritogenesis and survival. On the other hand, activation of MAPK pathway contributes only to neuritogenesis, but not survival [37]. Indeed, PI3K pathway is known to promote oncogenesis via inhibition of apoptosis as one of the mechanisms [52]. Both activation of PI3K pathway and over-expression of TrkB and BDNF are associated with poor prognosis in NB [13,53,54]. In contrast, patients with increased levels of TrkA and TrkC demonstrate better prognosis [55,56]. Thus our results imply that *HOXD-AS1 *may be regulated via PI3K pathway induced by oncogenic BDNF/TrkB axis. This is further supported by our observation that HOXD-AS1 transcript positively correlates with TrkB and negatively correlates with TrkA and TrkC (Additional file 16**panel B**).

In agreement with the proposed pro-oncogenic role of HOXD-AS1, we observed that its siRNA- mediated knock-down resulted in differential expression of genes implicated in angiogenesis and inflammation, two highly interdependent processes considered as the hallmarks of cancer [29]. Both of these processes are manifested in a crosstalk between tumor cells and their microenvironment that includes endothelial and immune inflammatory cells. In agreement with this, a number of HOXD-AS1 target genes encode secreted proteins that provide communication between tumor and its microenvironment. Among them are cytokines, such as CX3CL1, CCL20, TNF, GDF15 - key regulators of angiogenesis and lymphangiogenesis ANG and PROX1 - genes involved in remodeling of extracellular matrix LOX and ADAMTS3.

## Conclusions

In conclusion, the computational pipeline developed in this study was successfully applied to greatly extend the number of novel NB cancer biomarkers using published Affymetrix U133 chip datasets and to identify candidate lncRNAs functionally implicated in classification of the NB subtypes. We found that one of these lncRNAs, named HOXD-AS1, is induced by RA, could be regulated via PI3K/Akt pathway, controls genes involved in RA signaling, angiogenesis and inflammation.

## Methods

### Cell culture

SH-SY5Y cell line was purchased from American Type Culture Collection (ATCC). SH-SY5Y cells were cultured in Hams F12/ DMEM supplemented with 10% FBS in a humidified incubator at 37^0^C with 5% CO_2_. For cell differentiation experiment SH-SY5Y cells were plated onto laminin coated dishes and on the next day were induced to differentiate by addition of 10 µM all-trans-retinoic acid (RA, Sigma). For pathway inhibition study SH-SY5Y were induced to differentiate by 10 µM in the absence or presence of 10 µM of LY294002, PD98059 and SB203580 (Sigma).

### RNA isolation

Total RNA was purified using Trizol. For microarray experiment RNA was additionally purified using RNeasy Mini Kit (Qiagen) according to manufacturer's instructions.

### Northern blot

The probe to detect HOXD-AS1 was designed to be of 373 nucleotides, mapping to coordinates chr2:177,040,872-177,041,244 in GRCh37/hg19 Human Assembly of UCSC browser. The probe was synthesized (using cDNA derived from SH-SY5Y total RNA as a template) and Northern blot procedures were performed using DIG Northern Starter Kit according to manufacturer's instructions.

### Quantitative PCR (qRT-PCR)

Total RNA was used as a template for reverse transcription using QuantiTect Reverse Transcription Kit (Qiagen) using random hexamer primers. The transcript levels were analyzed by qRT-PCR run on Rotor-Gene Q machine using Rotor-Gene SYBR Green PCR Kit (Qiagen). The primers used throughout this study are listed in Additional file 17.

### Knock-down experiment using small interfering RNA (siRNA)

siRNA duplexes and control ON-TARGETplus non-targeting pool (Dharmacon) were introduced into SH-SY5Y cells using AmaxaNucleofector System (Lonza AG) according to manufacturer"s instructions. Cells were plated onto laminin coated dishes and 24 hours later were induced to differentiate with 10 µM RA. 48 hours later cells were harvested for RNA analysis. The following siRNA target sequences (Dharmacon) were used to knock-down HOXD-AS1: GAAAGAAGGACCAAAGTAA, GCACAAAGGAACAAGGAAA.

### Microarray analysis

Total RNA was amplified using IlluminaTotalPrep RNA Amplification Kit (Ambion) and amplified cRNA was hybridized to HumanHT-12 v4 BeadChip according to manufacturer"s instructions. Briefly, 500 ng of total RNA was converted to double stranded cDNA using T7-oligo(dT) primers followed by IVT reaction to produce biotinylatedcRNA. The chips were scanned on IlluminaBeadArray Reader. The microarray data were deposited to NCBI GSE40680.

### Analysis of microarray data from knock-down experiments

Expression values from four technical replicates were analyzed for each of the three biological samples (two siRNA knock-down experiments and one non-matching siRNA control). After RMA normalization, microarray signal distributions were examined. Multiple statistical criteria were used to obtain robust results of differential expression analysis. A differentially expressed probe simultaneously satisfied each of the following criteria: 1) Student's t-test P(sample vs. control) <0.05, 2) fold change ratio of median sample value to median control value > 1.3 or >1.4, 3) the number of sample replicas which signal value is higher than median control value is not less than 3. Based on these criteria 98 differentially expressed probes were selected.

### Computational pipeline to define and classify probe sets for lncRNAs on Affymetrix U133- Plus-2.0

Noncoding status of the probe sets was confirmed by assuring that their sequences share no homology with existing protein-coding genes with NCBI BLAST [32]. To further confirm that the selected transcripts were not protein-coding by the base composistion statistical criteria, CRITICA software was used as an additional filter [31]. The overlap length distribution of the probe sets and the noncoding transcripts was calculated and parametrized with a 3-component mixture model, a sum of 3 extended power-law distribution functions by least squares method [57]. To generate a high-confidence subset of lncRNA genes used in the study, the all Affymetrix U133-Plus-2.0 probes were matched against Gencode v.19 database of lncRNA transcripts using NCBI BLAST tool with the following options:-F F -g F -a 4 -K 1 -e 0.001.

### Computational analysis of clinical microarray data

For identification of lncRNAs differentially expressed in neuroblastoma, GSE12460 [58] AffymetrixU133-Plus-2.0 data from GEO database were used. This dataset contains 64 neuroblastoma (stages 1 to 4) tumor samples, in the genomes of 14 of which MYCN gene was amplified. For clinical analysis, lncRNA longer than 200 bp were selected. Mann-Whitney U test with null-hypothesis that the expression of a given lncRNA in the samples of neuroblastoma patients diagnosed at stages I or II is distributed equivalently to the patients diagnosed at stages III or IV and Student's t-test with null hypothesis that the population means are equal were applied with FDR control [59]. For every lncRNA Kendall's tau correlation was calculated across the patients sorted by their diagnosed neuroblastoma stages (I to IV). To study relapsing neuroblastoma tumors 102 patients of GSE3446 dataset [13] was used. To study the effect of RNA gene expression on 5-years patient survival 44 patients of GSE16237 dataset [60] was used. Mann-Whitney U test for assessing gene differential expression and PAM analysis [61] for gene signature identification were used for these two datasets.

### GO analysis

RefSeq genes located in the vicinity of +/-10 kb from each studied lncRNAs were defined as neighboring. For each neighboring gene, the best matching Affymetrix probe set was found, according to APMA annotation (30). For each such probe set, the best matching mRNA was identified. For all such mRNAs found for all neighboring genes Entrez IDs were found according to UniProt annotation. For each Entrez ID, the corresponding GO were found from Panther database (http://www.pantherdb.org/). Enrichment was considered significant for ontologies with P < 0.01 and enrichment value not less than 2.

### Fluorescence in situ hybridization

RNA visualization was performed using QuantiGeneViewRNA technology (Panomics). RNA staining was performed according to manufacturer's protocol. The cells were counterstained with DAPI to label nuclei and images were acquired on Zeiss LSM 5 DUO confocal microscope.

## Authors' contributions

AAY, AOB, VAK, IVK planned the project. AAY, VAK, IVK designed the experiments. AAY, JZT, GMS, PS, IVK performed experiments. AOB, VAK performed computational analysis. AAY, AOB, VAK, IVK wrote the paper. All authors discussed the data and commented on the manuscript.

## Conflict of interests

The authors declare that they have no competing interests.

## Supplementary Material

Additional file 1 **Classification of AffymetrixU133 probe sets representing ncRNAs**. Click here for file

Additional file 2 **Length distribution of protein coding RNAs overlapping with Affymetrix U133 probe sets**. A) Histogram of length distribution of all protein coding RNAs represented on AffymetrixU133 arrays; green line represents the mixed model based on Gaussian components, red line represents the tail-specific component. B) Inverse cumulative distribution (iCDF) of the ncRNA length plotted in double log coordinates reveals that log-normal distribution (black solid curve) can describe it well and three-component (blue, green and cyan dashed curves respectively) mixture model (yellow solid curve) is unnecessary; each component of the mixture model is parametrized with a polynomial equation (coefficients a0, a1 and a2). Click here for file

Additional file 3 **Histogram of the ncRNAs (represented by Affymetrix U133 probe sets) length distribution by chromosome**. Blue and yellow parts of the bar respectively represent the number of ncRNAs, which genes are with and without neighbouring genes within 10 kb. Click here for file

Additional file 4 **List of ncRNA probe sets neighboring coding genes with their ontologies**. Click here for file

Additional file 5 **lncRNAs with expression levels correlating with the disease stage**. A. List of 164 lncRNAprobe sets associated with neuroblastoma progression. B. NcRNA gene expression signature for discriminating aggressive (stage III-IV) from not agressive (stage I-II) neuroblastoma. C.Predictive power of the 159 ncRNA gene expression signature for classification of neuroblastoma tumors into aggressive (stages III-IV) and low-aggressive (stage I-II) cases. D. NcRNA gene expression signature for discriminating primary neuroblastoma tumors of patients with relapse vs. tumors of patients without relapse. E. Predictive power of the 159 ncRNA gene expression signature for classification of neuroblastoma tumors into aggressive (stages III-IV) and low-aggressive (stage I-II) cases. F. The 159 ncRNAs of the PAM signature for separating high- and low- aggressive forms of neuroblastoma form 2 major clusters. G. 159 lncRNAs are associated with neuroblastoma progression. Click here for file

Additional file 6 **Cluster analysis of Kendall's correlation matrix of 159 ncRNAs**. Cluster analysis revealed two major groups of correlating ncRNAs (left panel, red and green) characterized with specific GOs. Click here for file

Additional file 7 **Correlation of HOXD-AS1 expression with neuroblastoma progression**. Expression of HOXD-AS1 correlates with neuroblastoma progression and differentiates between early (st. 1-2) and late (st. 3-4) stage tumor belonging to A) non-MNA and B) MNA groups. Click here for file

Additional file 8 **UCSC browser representation of the locus between HoxD1 and HoxD3 genes**. Custom tracks depict positions of siRNAs used to knock down HOXD-AS1, pairs of primers targeting different parts of annotated transcripts within this locus (Locus primers and Exon 3 primers), HOXD-AS1 model, the tracks of transcript predictions (UCSC Genes, and Affymetrix U133 Plus probe sets). Two transcription start region defined by CpG Islands, ENCODE H3K27Ac mark and Transcription Factor ChIP-Seq tracks are shown in rectangles. Click here for file

Additional file 9 **The 3' exon of HOXD-AS1 is full length**. A set of primers targeting different regions of the entire length of HOXD-AS1 exon 3 was used to measure A) relative expression levels of these regions B) the knock-down efficiency of these regions by 2 indicated siRNAs (Additional file 14). C) Northern blot confirming the size of HOXD-AS1. Click here for file

Additional file 10 **Correlation of HOXD-AS1 and HOXD3 expression in patient tumors**. Expression of HOXD-AS1 transcript positively correlates (Kendall's tau) with expression of HoxD3 measured by probe sets 206601_at (A) and 206602_at (B) in non-MNA patient tumors. Click here for file

Additional file 11 **Identification of differentially expressed genes after knock-down of HOXD-AS1 in SH-SY5Y cells**. (A) SH-SY5Y cells grown on laminin coated dishes were transfected with non- targeting siRNA pool and two siRNAs targeting different regions of HOXD-AS1 transcript. On the next day transfected cells were induced to differentiate by addition of 10 µM RA. 48 hours later RNA was extracted and the efficiency of knock-down was estimated by qRT-PCR and expressed as a fold change relative to non-targeting siRNA pool (control). (B) SH-SY5Y cells were transfected with non-targeting siRNA pool and two siRNAs targeting different regions of HOXD-AS1 transcript. On the next day transfected cells were induced to differentiate by addition of 10 µM RA. 48 hours later RNA was extracted and the relative levels of the selected transcripts were estimated by qRT-PCR. Click here for file

Additional file 12 **List of 96 genes differentially regulated by HOXD-AS1 knock-down**. Click here for file

Additional file 13 **Genes regulated by HOXD-AS1 on expression level differ significantly in predicting relapse of neuroblastoma**. Expression distribution comparisons. Click here for file

Additional file 14 **HOXD-AS1 target genes involved in NB progression**. A. 12 of the 16 relapse-significant gene targets of HOXD-AS1 belong to RA pathway. B. HOXD-AS1 target genes, whose expression forms a signature discriminating between relapsing and not relapsing neuroblastoma tumors C. Predictive power of the 98 HOXD-AS1 target genes expression signature for classification of neuroblastoma tumors into relapsing and not relapsing cases. Click here for file

Additional file 15 **HOXD-AS1 is evenly distributed between nucleus and cytoplasm**. Subcellular localization of HOXD-AS1 lncRNA was analyzed by two methods. (A) SH-SY5Y cells were fractionated into cytoplasm and nucleus by centrifugation through sucrose pad (see Materials and Methods). RNA was extracted from nuclear and cytoplasmic fractions and the equal volumes of nuclear and cytoplasmic RNA, corresponding to equivalent numbers of cells were used for the first strand cDNA synthesis. The abundance of HOXD-AS1 transcript in nuclear fraction relative to cytoplasmic was analysed by qRT-PCR. The *bona fide *nuclear transcript MALAT1 and mitochondrial 16S were used to assess the purity of fractionation. (B) Localization of HOXD-AS1 inside SH-SY5Ywas directly visualized by hybridization of the specific PanomicsQuantiGeneViewRNA probes followed by confocal microscopy (see Materials and Methods). HOXD-AS1 transcript was visualized at red channel, cytoplasmic GAPDH transcript was visualized at green channel and nuclei were counter-stained with DAPI (blue). Click here for file

Additional file 16 **Correlation of HOXD-AS1 with TrkA, TrkB and TrkC receptors expression**. (A) qRT-PCR demonstrating drastic up-regulation of TrkB, but not TrkA and TrkC in the time-course of differentiation of SH-SY5Y cells. (B)HOXD-AS1 positively correlates with TrkB and negatively correlates with TrkA and TrkC. Click here for file

Additional file 17 **Primer list**. Click here for file

## Declarations

Publication of this article was funded by Bioinformatics Institute, Agency for Science, Technology and Research (A*STAR), Singapore.

This article has been published as part of *BMC Genomics *Volume 15 Supplement 9, 2014: Thirteenth International Conference on Bioinformatics (InCoB2014): Computational Biology. The full contents of the supplement are available online at http://www.biomedcentral.com/bmcgenomics/supplements/15/S9.
