# Supplementary figures and images for: HOXD-AS1 is a novel lncRNA encoded in HOXD cluster and a marker of neuroblastoma progression revealed via integrative analysis of noncoding transcriptome
Source: BMC Genomics. 2014 Dec 8;15(Suppl 9):S7. doi: 10.1186/1471-2164-15-S9-S7 (PMC4290621; doi:10.1186/1471-2164-15-S9-S7)

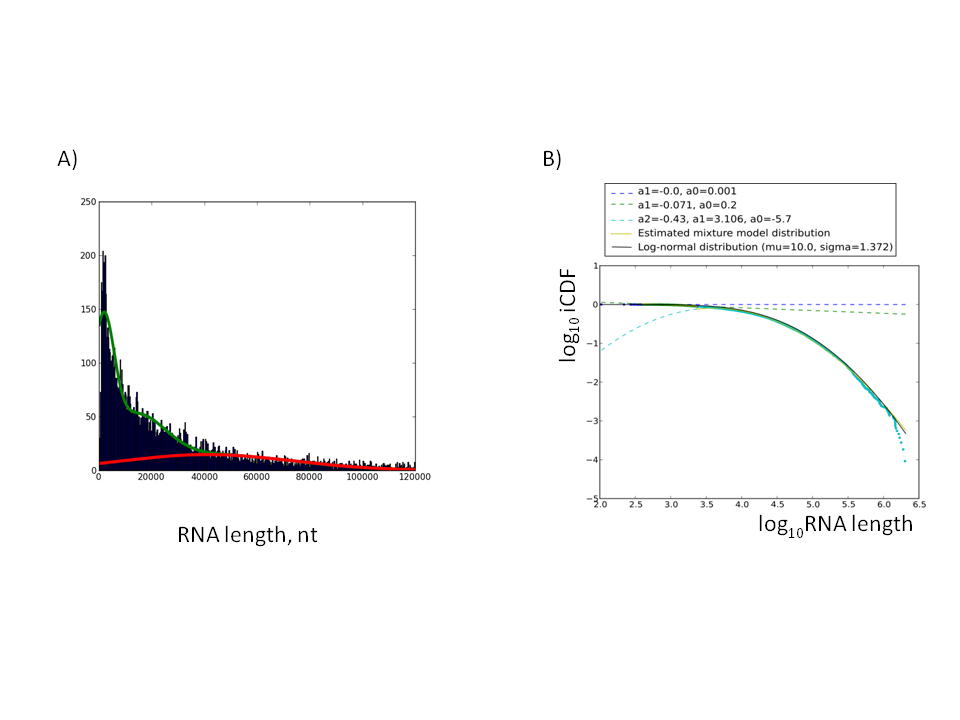

Supplement: Additional file 2 — Length distribution of protein coding RNAs overlapping with Affymetrix U133 probe sets. A) Histogram of length distribution of all protein coding RNAs represented on AffymetrixU133 arrays; green line represents the mixed model based on Gaussian components, red line represents the tail-specific component. B) Inverse cumulative distribution (iCDF) of the ncRNA length plotted in double log coordinates reveals that log-normal distribution (black solid curve) can describe it well and three-component (blue, green and cyan dashed curves respectively) mixture model (yellow solid curve) is unnecessary; each component of the mixture model is parametrized with a polynomial equation (coefficients a0, a1 and a2). [file 1471-2164-15-S9-S7-S2.TIF]

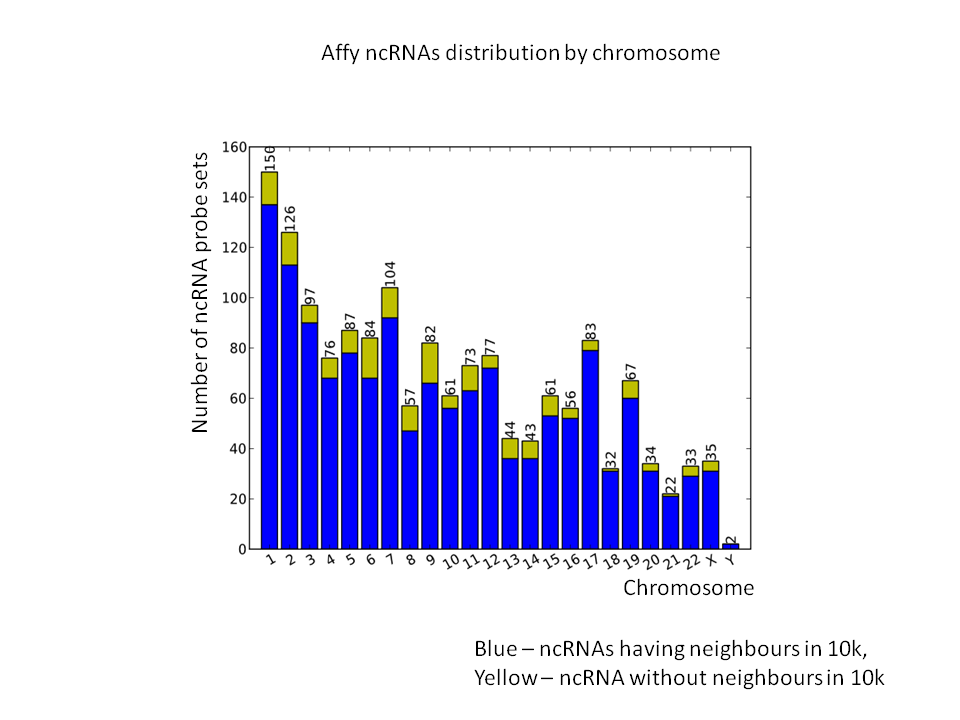

Supplement: Additional file 3 — Histogram of the ncRNAs (represented by Affymetrix U133 probe sets) length distribution by chromosome. Blue and yellow parts of the bar respectively represent the number of ncRNAs, which genes are with and without neighbouring genes within 10 kb. [file 1471-2164-15-S9-S7-S3.TIF]

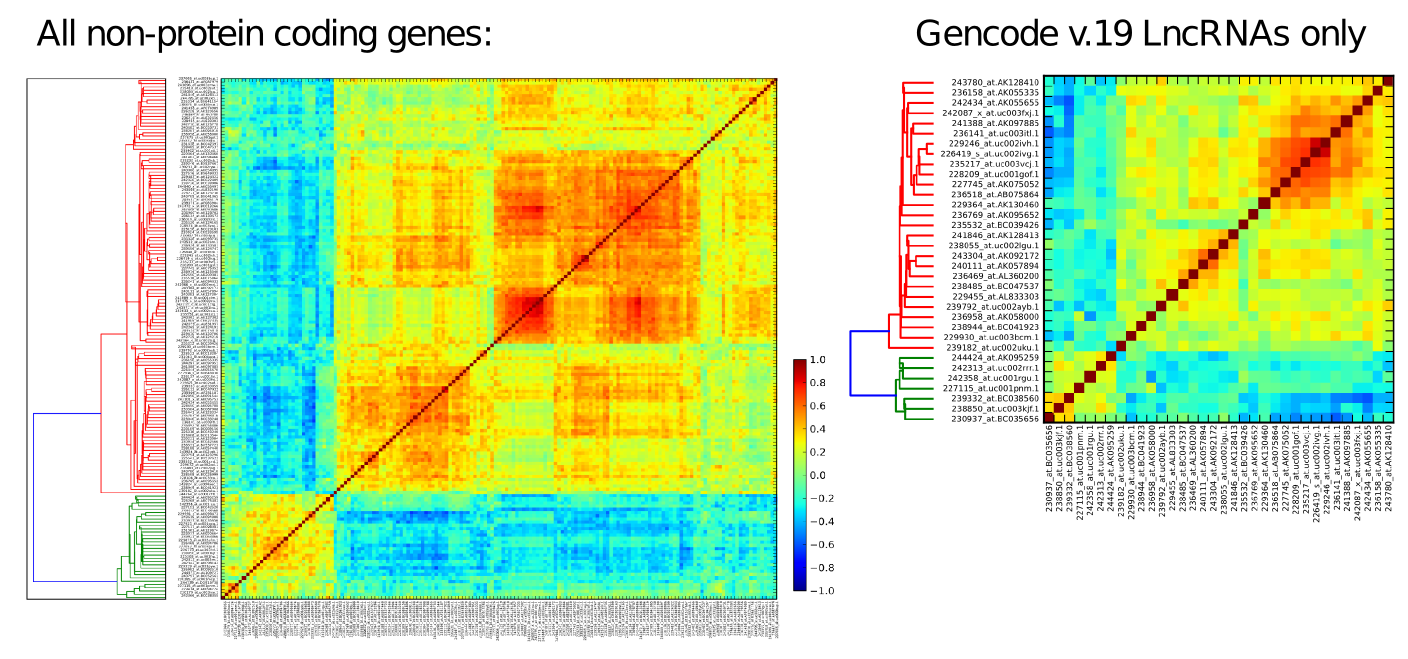

Supplement: Additional file 6 — Cluster analysis of Kendall's correlation matrix of 159 ncRNAs. Cluster analysis revealed two major groups of correlating ncRNAs (left panel, red and green) characterized with specific GOs. [file 1471-2164-15-S9-S7-S6.tif]

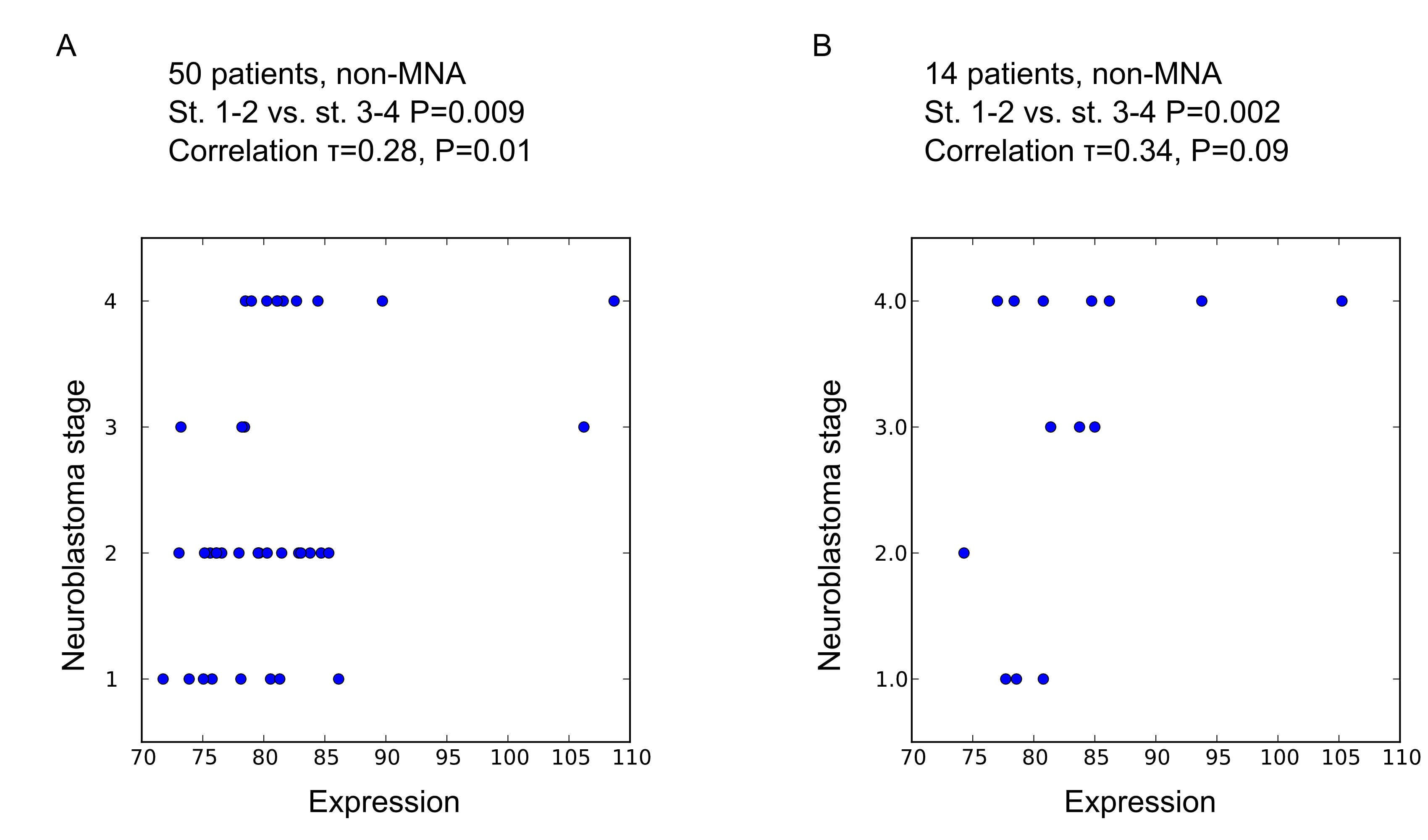

Supplement: Additional file 7 — Correlation of HOXD-AS1 expression with neuroblastoma progression. Expression of HOXD-AS1 correlates with neuroblastoma progression and differentiates between early (st. 1-2) and late (st. 3-4) stage tumor belonging to A) non-MNA and B) MNA groups. [file 1471-2164-15-S9-S7-S7.tif]

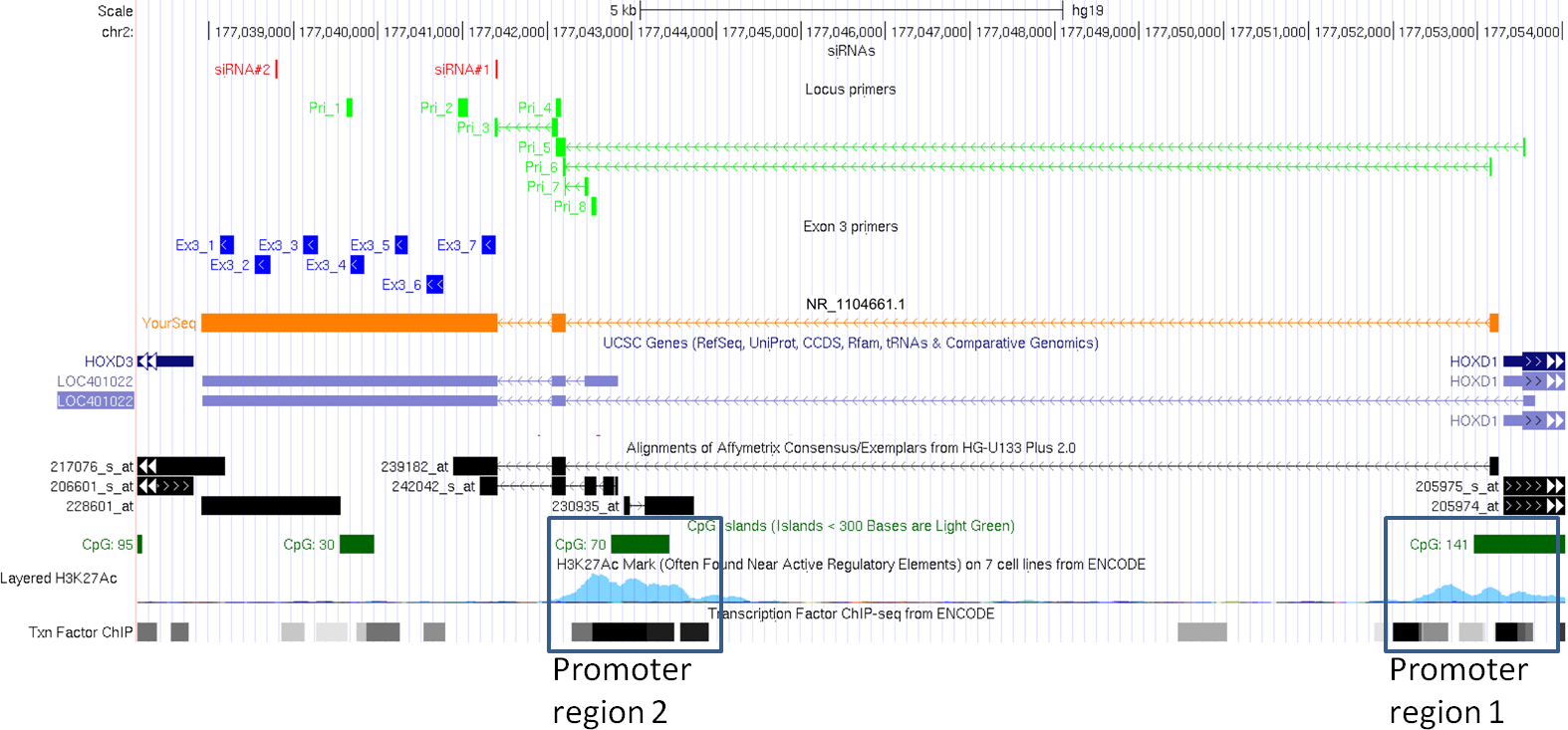

Supplement: Additional file 8 — UCSC browser representation of the locus between HoxD1 and HoxD3 genes. Custom tracks depict positions of siRNAs used to knock down HOXD-AS1, pairs of primers targeting different parts of annotated transcripts within this locus (Locus primers and Exon 3 primers), HOXD-AS1 model, the tracks of transcript predictions (UCSC Genes, and Affymetrix U133 Plus probe sets). Two transcription start region defined by CpG Islands, ENCODE H3K27Ac mark and Transcription Factor ChIP-Seq tracks are shown in rectangles. [file 1471-2164-15-S9-S7-S8.tif]

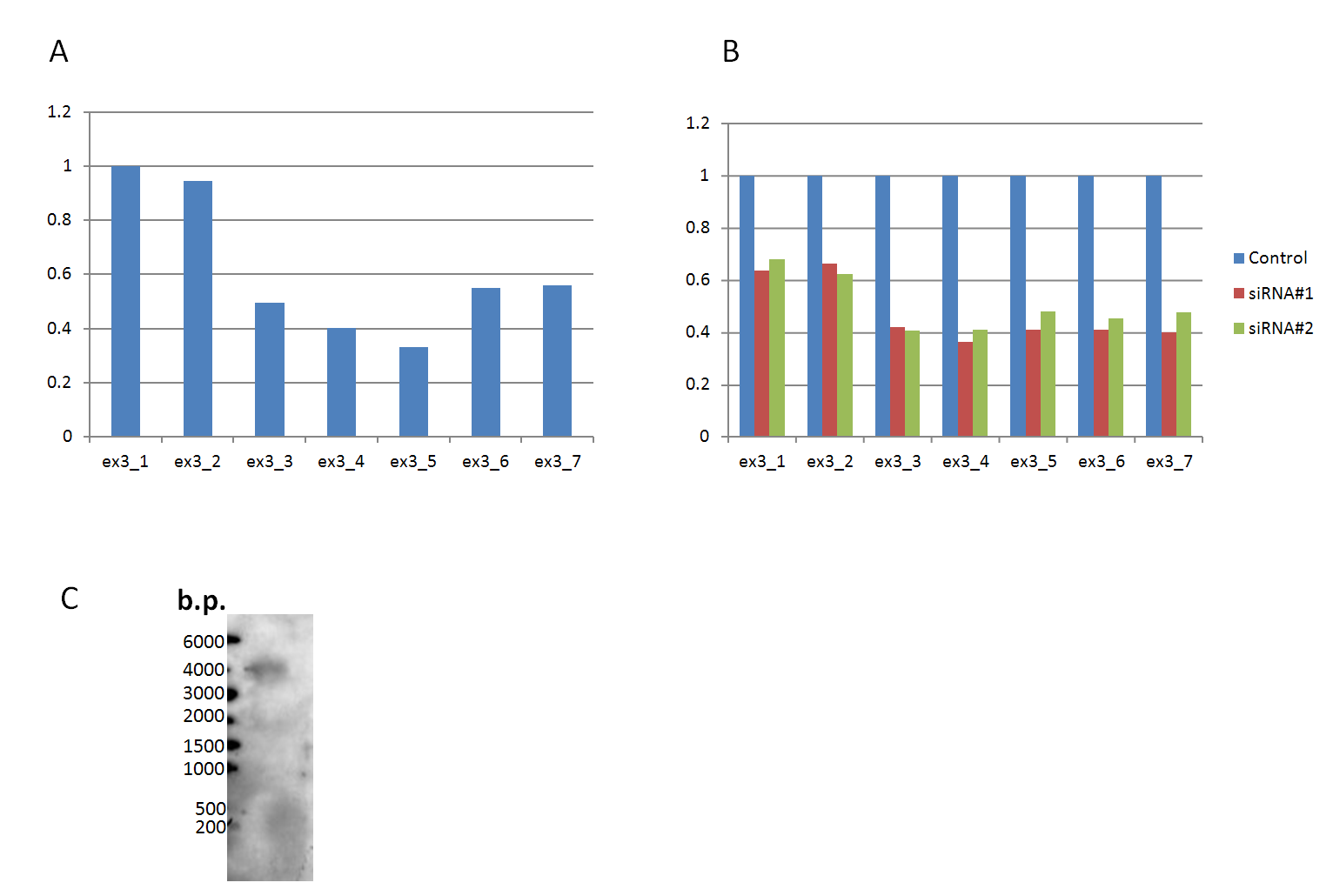

Supplement: Additional file 9 — The 3' exon of HOXD-AS1 is full length. A set of primers targeting different regions of the entire length of HOXD-AS1 exon 3 was used to measure A) relative expression levels of these regions B) the knock-down efficiency of these regions by 2 indicated siRNAs (Additional file 14). C) Northern blot confirming the size of HOXD-AS1. [file 1471-2164-15-S9-S7-S9.tif]

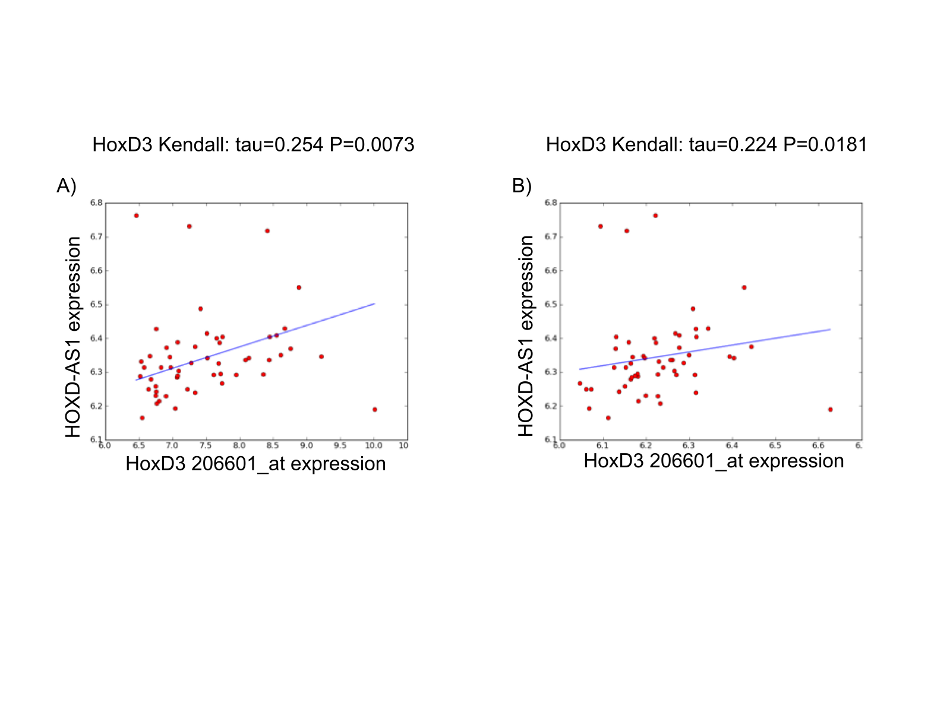

Supplement: Additional file 10 — Correlation of HOXD-AS1 and HOXD3 expression in patient tumors. Expression of HOXD-AS1 transcript positively correlates (Kendall's tau) with expression of HoxD3 measured by probe sets 206601_at (A) and 206602_at (B) in non-MNA patient tumors. [file 1471-2164-15-S9-S7-S10.tif]

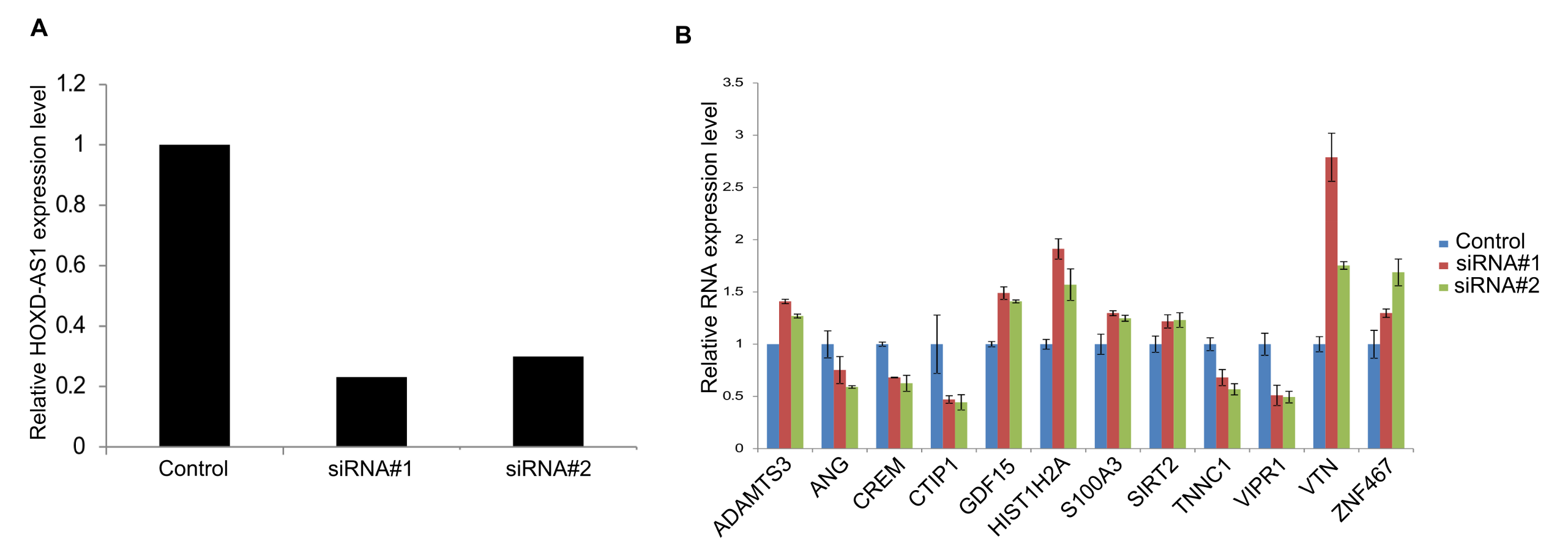

Supplement: Additional file 11 — Identification of differentially expressed genes after knock-down of HOXD-AS1 in SH-SY5Y cells. (A) SH-SY5Y cells grown on laminin coated dishes were transfected with non- targeting siRNA pool and two siRNAs targeting different regions of HOXD-AS1 transcript. On the next day transfected cells were induced to differentiate by addition of 10 µM RA. 48 hours later RNA was extracted and the efficiency of knock-down was estimated by qRT-PCR and expressed as a fold change relative to non-targeting siRNA pool (control). (B) SH-SY5Y cells were transfected with non-targeting siRNA pool and two siRNAs targeting different regions of HOXD-AS1 transcript. On the next day transfected cells were induced to differentiate by addition of 10 µM RA. 48 hours later RNA was extracted and the relative levels of the selected transcripts were estimated by qRT-PCR. [file 1471-2164-15-S9-S7-S11.tif]

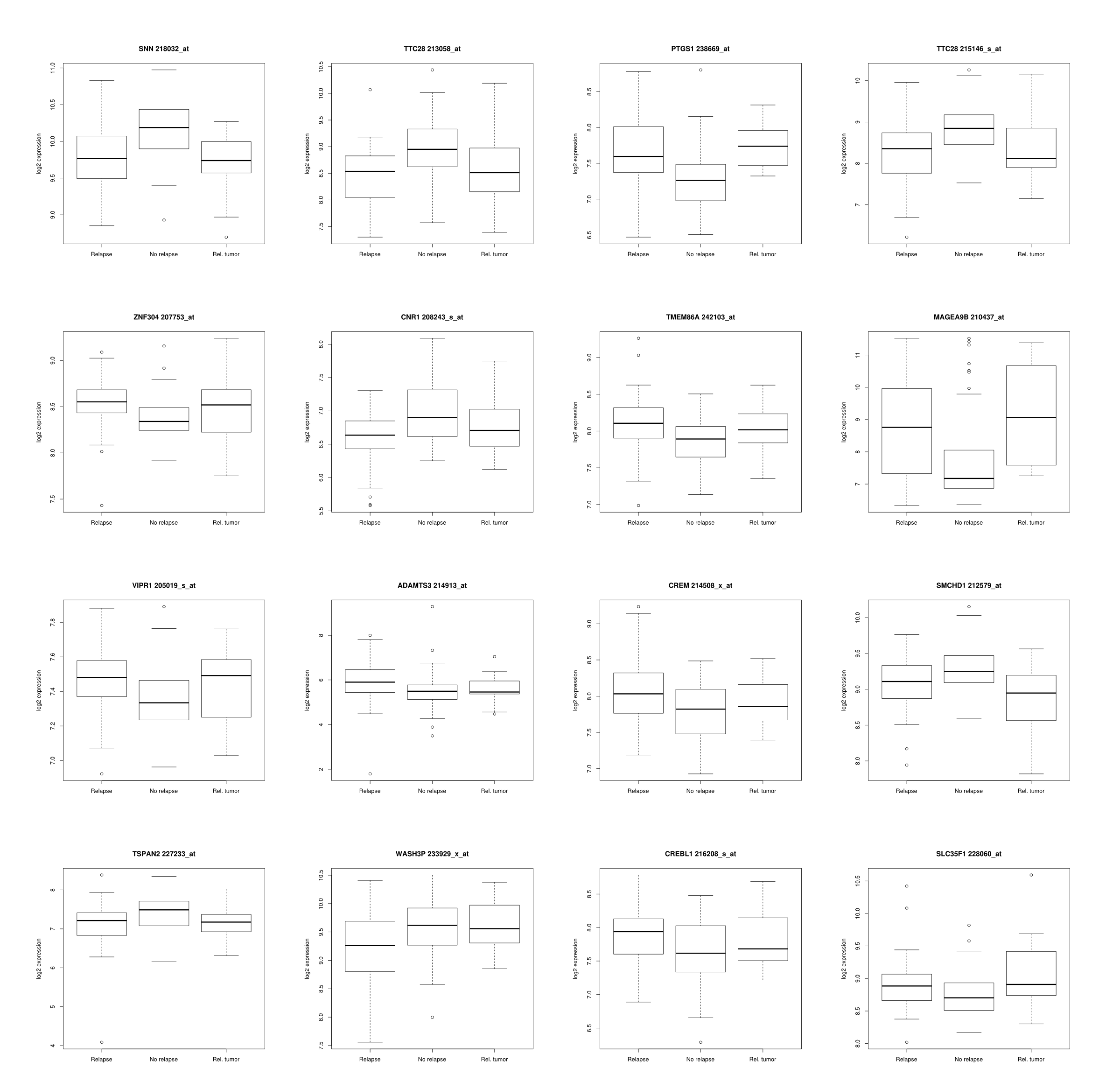

Supplement: Additional file 13 — Genes regulated by HOXD-AS1 on expression level differ significantly in predicting relapse of neuroblastoma. Expression distribution comparisons. [file 1471-2164-15-S9-S7-S13.tif]

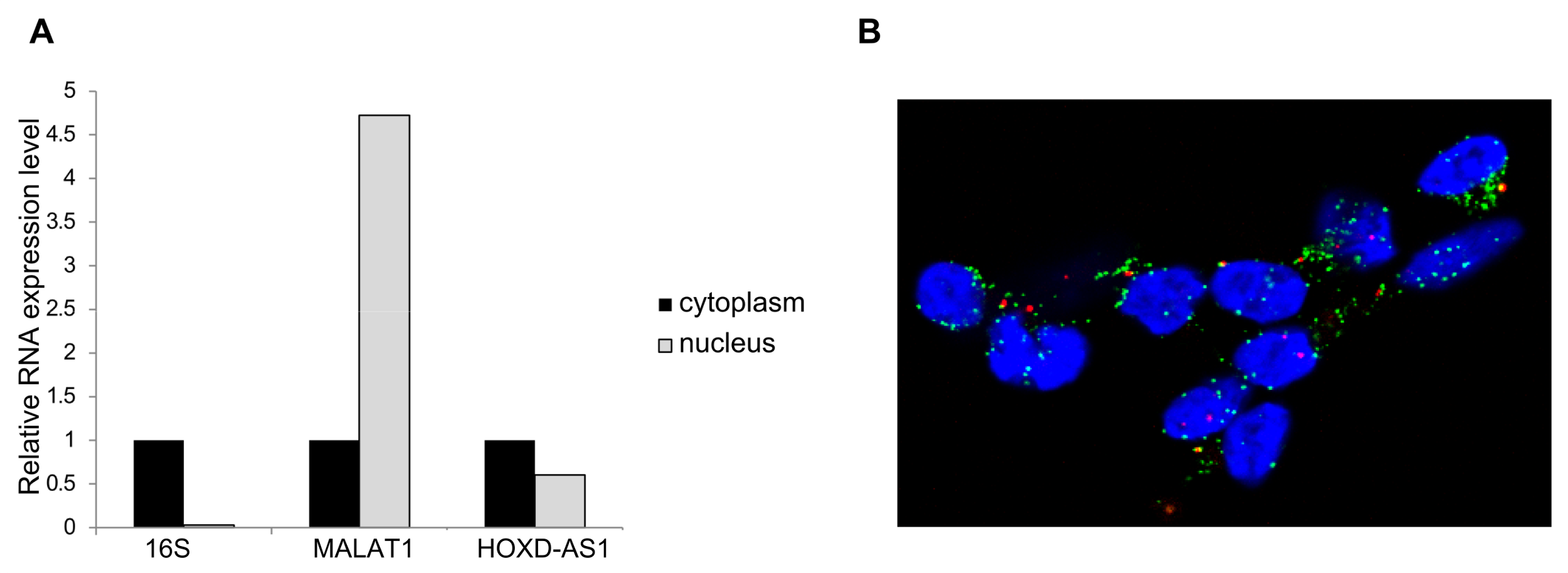

Supplement: Additional file 15 — HOXD-AS1 is evenly distributed between nucleus and cytoplasm. Subcellular localization of HOXD-AS1 lncRNA was analyzed by two methods. (A) SH-SY5Y cells were fractionated into cytoplasm and nucleus by centrifugation through sucrose pad (see Materials and Methods). RNA was extracted from nuclear and cytoplasmic fractions and the equal volumes of nuclear and cytoplasmic RNA, corresponding to equivalent numbers of cells were used for the first strand cDNA synthesis. The abundance of HOXD-AS1 transcript in nuclear fraction relative to cytoplasmic was analysed by qRT-PCR. The bona fide nuclear transcript MALAT1 and mitochondrial 16S were used to assess the purity of fractionation. (B) Localization of HOXD-AS1 inside SH-SY5Ywas directly visualized by hybridization of the specific PanomicsQuantiGeneViewRNA probes followed by confocal microscopy (see Materials and Methods). HOXD-AS1 transcript was visualized at red channel, cytoplasmic GAPDH transcript was visualized at green channel and nuclei were counter-stained with DAPI (blue). [file 1471-2164-15-S9-S7-S15.tif]

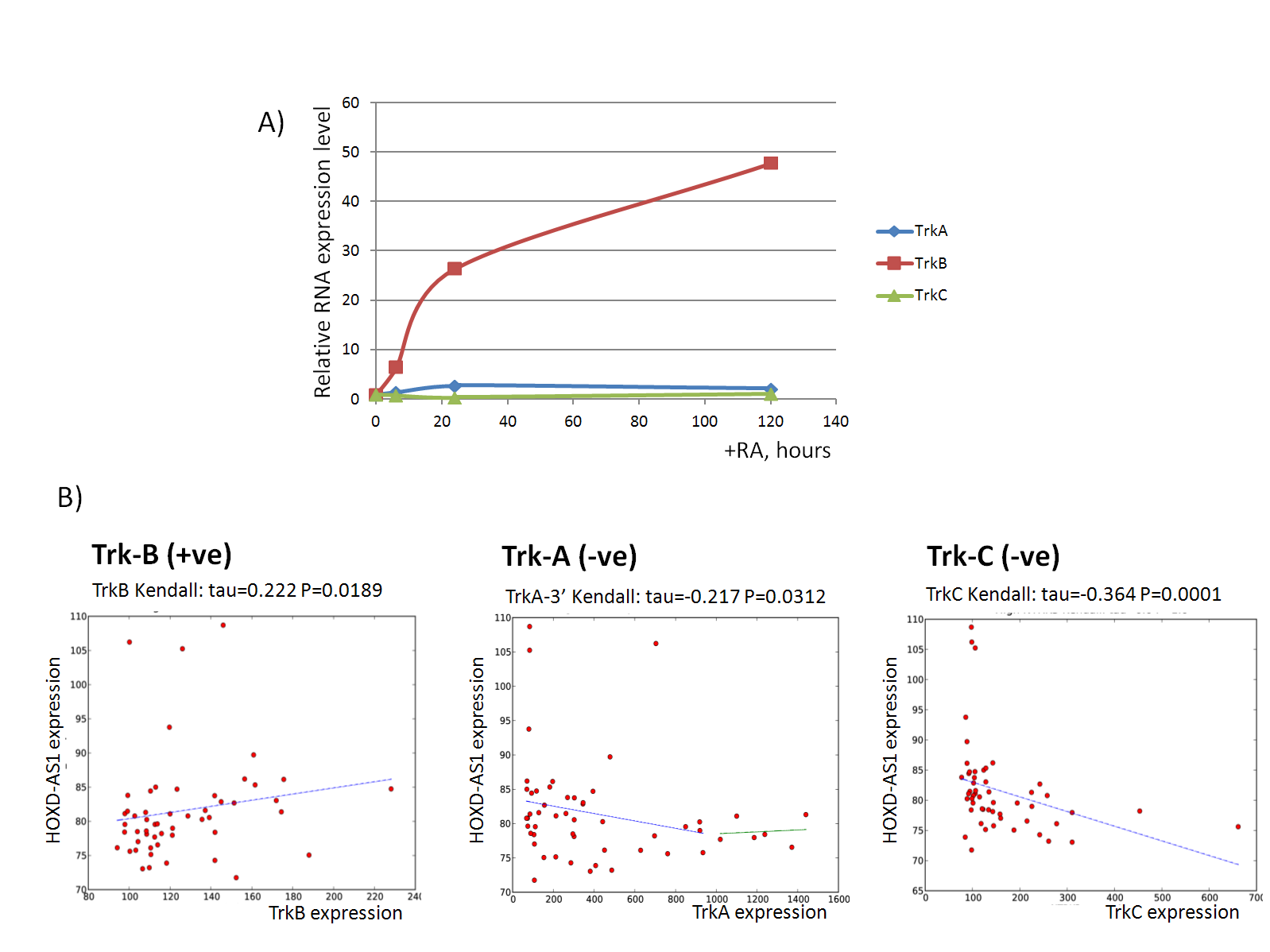

Supplement: Additional file 16 — Correlation of HOXD-AS1 with TrkA, TrkB and TrkC receptors expression. (A) qRT-PCR demonstrating drastic up-regulation of TrkB, but not TrkA and TrkC in the time-course of differentiation of SH-SY5Y cells. (B)HOXD-AS1 positively correlates with TrkB and negatively correlates with TrkA and TrkC. [file 1471-2164-15-S9-S7-S16.tif]
